# A New Computational Model for Neuro-Glio-Vascular Coupling: Astrocyte Activation Can Explain Cerebral Blood Flow Nonlinear Response to Interictal Events

**DOI:** 10.1371/journal.pone.0147292

**Published:** 2016-02-05

**Authors:** Solenna Blanchard, Sandrine Saillet, Anton Ivanov, Pascal Benquet, Christian-George Bénar, Mélanie Pélégrini-Issac, Habib Benali, Fabrice Wendling

**Affiliations:** 1 Université de Rennes 1, INSERM U1099, Laboratoire Traitement du Signal et de l’Image, Rennes, France; 2 Aix Marseille Université, INSERM UMRS 1106, Institut de Neurosciences des Systèmes, Marseille, France; 3 Sorbonne Universités, UPMC Univ. Paris 06, CNRS, INSERM, Laboratoire d’Imagerie Biomédicale, Paris, France; University of Muenster, GERMANY

## Abstract

Developing a clear understanding of the relationship between cerebral blood flow (CBF) response and neuronal activity is of significant importance because CBF increase is essential to the health of neurons, for instance through oxygen supply. This relationship can be investigated by analyzing multimodal (fMRI, PET, laser Doppler…) recordings. However, the important number of intermediate (non-observable) variables involved in the underlying neurovascular coupling makes the discovery of mechanisms all the more difficult from the sole multimodal data. We present a new computational model developed at the population scale (voxel) with physiologically relevant but simple equations to facilitate the interpretation of regional multimodal recordings. This model links neuronal activity to regional CBF dynamics through neuro-glio-vascular coupling. This coupling involves a population of glial cells called astrocytes via their role in neurotransmitter (glutamate and GABA) recycling and their impact on neighboring vessels. In epilepsy, neuronal networks generate epileptiform discharges, leading to variations in astrocytic and CBF dynamics. In this study, we took advantage of these large variations in neuronal activity magnitude to test the capacity of our model to reproduce experimental data. We compared simulations from our model with isolated epileptiform events, which were obtained in vivo by simultaneous local field potential and laser Doppler recordings in rats after local bicuculline injection. We showed a predominant neuronal contribution for low level discharges and a significant astrocytic contribution for higher level discharges. Besides, neuronal contribution to CBF was linear while astrocytic contribution was nonlinear. Results thus indicate that the relationship between neuronal activity and CBF magnitudes can be nonlinear for isolated events and that this nonlinearity is due to astrocytic activity, highlighting the importance of astrocytes in the interpretation of regional recordings.

## Introduction

The dynamics of cerebral blood flow (CBF) changes are an essential element of neuronal environment, as they reflect nutriment supplies such as oxygen and glucose. Their link to neuronal activity, usually called neurovascular coupling, can now be investigated by multimodal recordings [1] such as simultaneous Local Field Potential (LFP)-laser Doppler (LD) recordings [2]. However, due to the multiple signaling pathways underlying this coupling, such multimodal data remain difficult to interpret.

**Fig 1:**
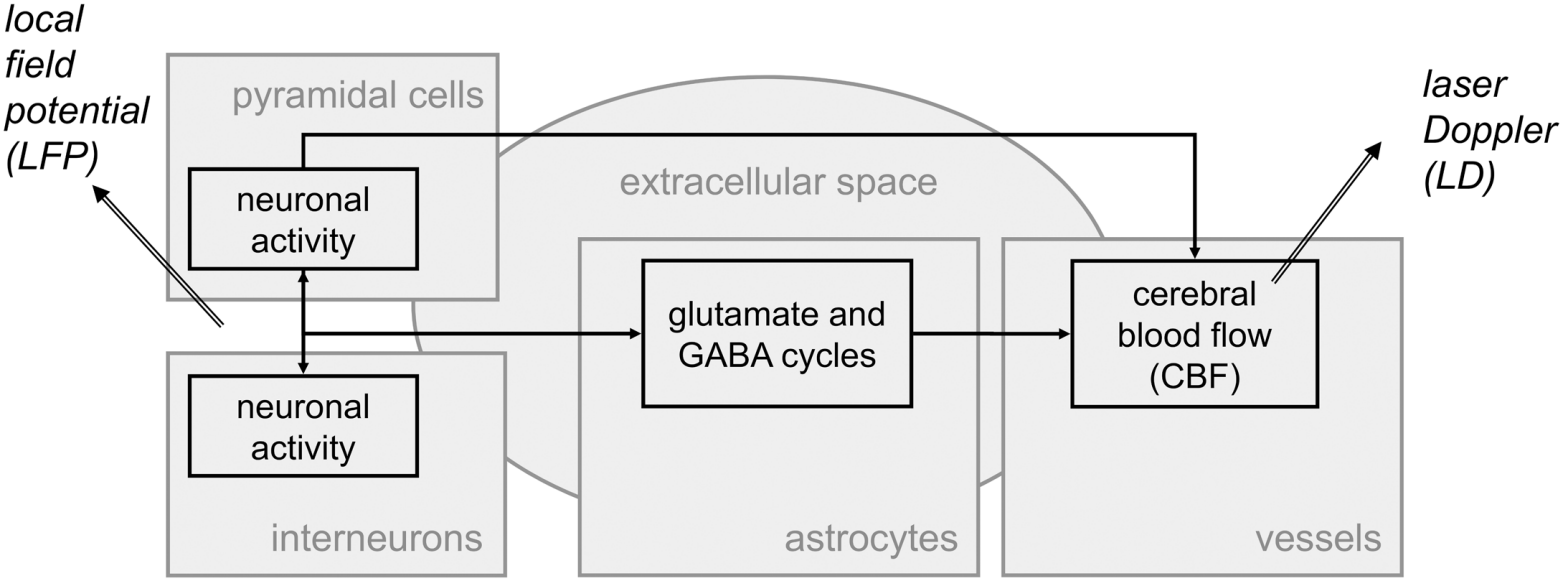
Main neuro-glio-vascular interactions in a voxel and their link to bimodal LFP-LD recordings. Arrows represent the interactions between the five compartments of the model: the compartments of pyramidal cells and interneurons provide a representation of the neuronal activity as measured by local field potential (LFP); the astrocytic compartment represents the key role of astrocytes in neurotransmitter (glutamate and GABA) cycling and cerebral blood flow (CBF) dynamics; the vessel compartment involves the CBF dynamics as measured by laser Doppler (LD); the extracellular space represents neurotransmitter exchanges between the other compartments.

Computational models can provide a key tool to this interpretation. For instance, the well-known model [3] representing CBF dynamics has been widely studied and used, including for data interpretation [4, 5]. In this model, CBF increase is driven by one neuronal input. Later, a number of studies have shown that astrocytes [6] also contribute to CBF increase, in parallel to a neuronal contribution [7, 8]. Actually, astrocytes have been shown to have a significant impact on either CBF dynamics [9] or metabolic regulation [10], although this impact cannot be measured easily. Computational models have the capacity to reveal different (patho-)physiological mechanisms [11] by comprising non-easily observable variables. Some computational studies have included a link to physiological literature, for instance by modeling neuronal-metabolic coupling through glucose and/or oxygen activities [12–16]. Nonetheless, these models do not account for astrocytes and lack essential details needed for interpreting patho-physiological mechanisms such as neurotransmitter exchanges. Actually, a comprehensive literature review (S1 File) showed that the cycles of glutamate and GABA (main neurotransmitters of the central nervous system) lie at the origin of astrocytic contribution to CBF changes. Some computational models describe pathways including glutamate and GABA transport and/or astrocytic activity [17–24], however these models deal with exchanges that occur at the cellular level of the tripartite synapse. Our goal was thus to conceive a new neuro-glio-vascular (NGV) model, representing the relationship between local neuronal activity (as measured by LFP) and CBF changes (as measured by LD) and introducing the contribution of astrocytes to CBF changes. Bimodal LFP-LD recordings (Fig 1) involve interactions between cell populations at the voxel scale (about 3 *mm*^*3*^). In the continuity of the pioneer work [25–27] expanded in [28–30] and introducing astrocytes in their metabolic role, we used a compartmental model (Fig 1) with an extracellular space to account for neurotransmitter exchanges. Since glutamate and GABA are primarily released by pyramidal cells and interneurons respectively, we distinguished between these two types of neuronal populations in the model.

Comparing a computational model and experimental data allows one to test the limitations of the model and its ability to reproduce physiological phenomena (choice of mathematical descriptions and incorporated variables, resulting complexity, see Materials and Methods). Simulations from our model were compared with isolated events extracted from simultaneous (LFP-LD) recordings (Materials and Methods) acquired in the context of epilepsy. More precisely, isolated events called epileptiform discharges occur in LFP signals after bicuculline injection in the cortex. These discharges elicit CBF events visible on LD data, the variations of which are sufficiently large to enable the study of the relationship between the magnitude of the discharges and that of the resulting CBF events. We obtained a good agreement between the model and the data according to the shape of the responses, which indicate that the model is able to reproduce epileptic phenomena. Besides, the sets of parameters found to obtain close simulated and experimental CBF magnitudes suggest that the relationship between neuronal activity and CBF response is nonlinear for epileptiform isolated discharges; and that this nonlinearity is due to the contribution of the astrocytes to CBF magnitude.

## Results

### Adaptation of a neural mass model to an animal acute model of epilepsy

**Fig 2:**
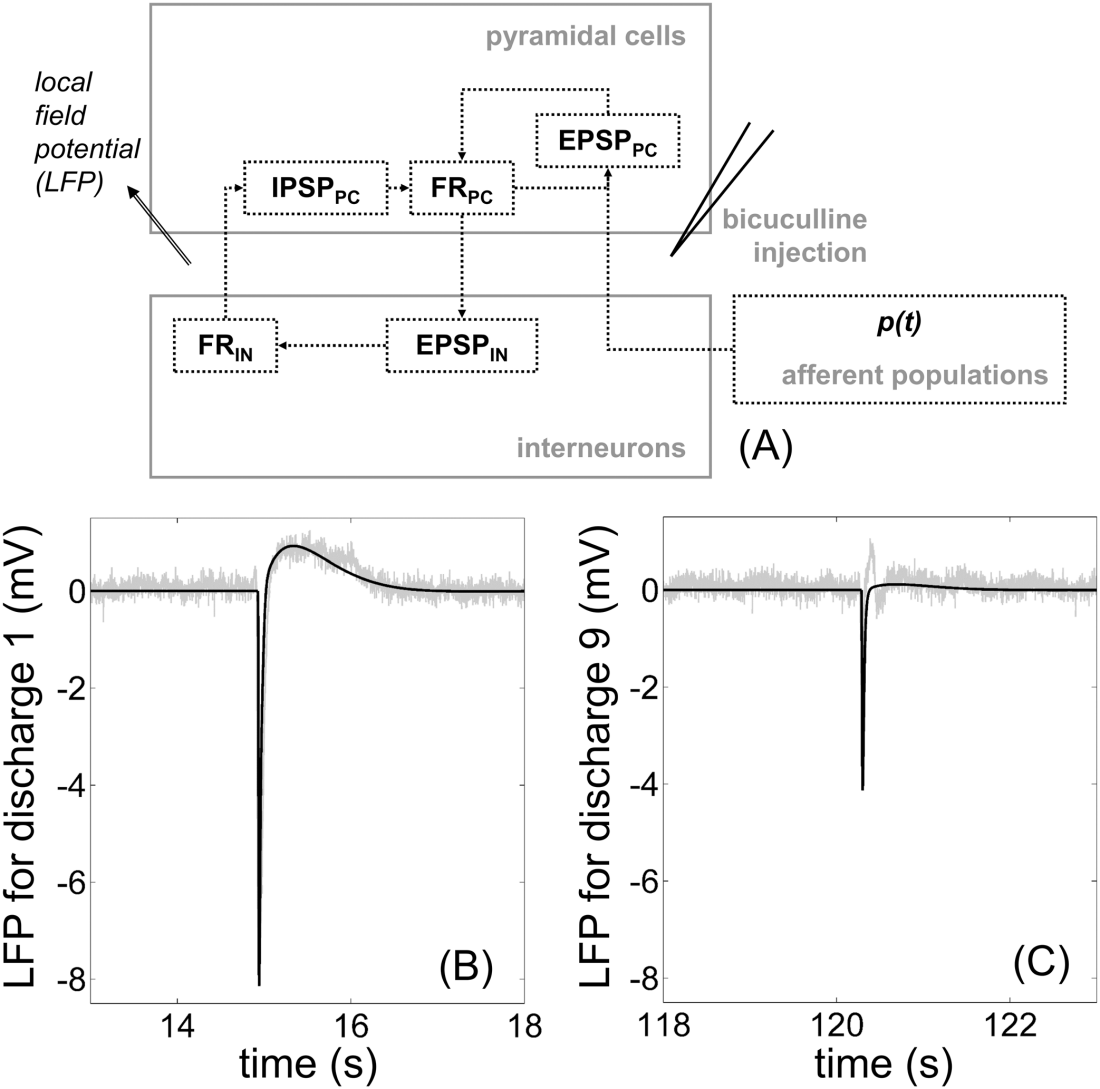
Modeling and experimental recording of neuronal activity. (A) Variables and relationships of the neural mass model (Table 1 and S1 Table). The input to the model is the activity coming from afferent populations, together with the injection of bicuculline in order to elicit an epileptic activity. The model output is the simulated local field potential (LFP). (B) and (C) Model-data comparison between the neural mass model (black) and LFP recording (light gray) for two isolated discharges. The mean value of *p* was set to *m*_*B*_ = 3.07 and its standard deviation to *σ*_*p*_ = 0. Magnitudes of the discharges (B) *A*_*peak*_ = 8.14 *mV* and (C) *A*_*peak*_ = 4.13 *mV* were obtained with the gain in Eq 2 set to (B) *G*_*1*_ = 965 and (C) *G*_*9*_ = 535.

The first part of the model concerns neuronal activity as measured by LFP (Fig 1). It is widely agreed that LFP can be interpreted as the sum of average excitatory and inhibitory post-synaptic potentials (*EPSP*_*PC*_ and *IPSP*_*PC*_) [31]. Firing rates from pyramidal cells and interneurons were also crucial variables (*FR*_*PC*_ and *FR*_*IN*_) to incorporate into the model as triggers to the release of glutamate and GABA (Fig 2A). These criteria have already been accounted for at the level of populations by a variety of neural mass models. We chose the model described in [32, 33]. These models are often used in epilepsy in order to study the deregulation of the excitation/inhibition balance.

In this context, the excitatory input *p* (Fig 2A) is usually defined as a Gaussian noise to represent the average density of afferent action potentials. Indeed, a Gaussian noise is able to induce spontaneous epileptiform discharges, which represent the epileptic activity induced by the pathology in experimental models where animals become epileptic, for instance after three weeks in the kainate mouse model [34]. Here, we relied on an acute model of epilepsy, namely epileptiform discharges elicited after injection of bicuculline (with injection site at 1000–1500 *μm*) in a healthy cortex (Materials and Methods), together with the effect of the afferent populations (LFP recorded at about 500 *μm*). Therefore, we described the input *p* to the model as a Gaussian noise with mean *m*_*p*_ and standard deviation *σ*_*p*_:

$$p(t)=Ν\left(m_{p},\sigma_{p}\right),$$ (1)

where

$$m_{p}=m_{B}+\sum_{i}G_{i}\left[H(t_{i})-H(t_{i}-\Delta )\right]$$ (2)

is the sum of the mean value *m*_*B*_ representing the effect of the afferent populations and a linear combination representing the effect of bicuculline injection (Fig 2A). In Eq 2, the effect of bicuculline injection is the rectangular function lasting Δ = 10 samples (i.e. 8 *ms* at 1250 *Hz*) and defined as the difference between the Heaviside function *H(t*_*i*_*)* at time *t*_*i*_, and its Δ-delayed version *H(t*_*i*_*-Δ)*. Gains *G*_*i*_ in Eq 2 represent the magnitude of the effect at time *t*_*i*_ and thus decreased over time as the magnitude of the discharges was reduced with bicuculline wash-out.

The average excitatory post-synaptic potential (from pyramidal cells to pyramidal cells, Fig 2A) was defined as:

$$\begin{array}{c} \frac{d^{2}EPSP_{PC}}{dt^{2}}=Aa\left[p(t)+C_{PC\to PC}.sigm\left(C_{PC\to IN}EPSP_{IN}(t),2e_{0},r_{N},s_{N}\right)\right]\\ -2a\frac{dEPSP_{PC}}{dt}-a^{2}EPSP_{PC}(t), \end{array}$$ (3)

where *EPSP*_*IN*_ is the average excitatory post-synaptic potential (from pyramidal cells to interneurons, Fig 2A) of interneurons. The sigmoid function in Eq 3 was defined as:

$$sigm(x,V,r,s)=\frac{V}{1+\text{exp}(rs-rx)}$$ (4)

where *x* is the dummy variable, *V* the maximum value, *s* the threshold and *r* the slope of the sigmoid (see Table 1 for a description of all the parameters used in the proposed NGV model, as well as the values either used in the literature or modified in the present study).

**Table 1:** Parameters values chosen from the experimental literature.

parameter name (unit)	physiological description	typical value	value in this article	references
*A (mV)*	average magnitude of excitatory post-synaptic potential	3 to 18	3.25	[32, 33, 35–39]
*1/a (ms)*	average time constant of excitatory post-synaptic potential at the dendrites of pyramidal cells	4.5 to 10	10	[32, 33, 35–39]
*B (mV)*	average magnitude of inhibitory post-synaptic potential	1 to 50	3	[32, 33, 35–39]
*1/b (ms)*	average time constant of inhibitory post-synaptic potential at the dendrites of pyramidal cells	20 to 70	400	[32, 33, 35–39]
*e*_*0*_ *(s*^*-1*^*)*	magnitude parameter of the neuronal sigmoid function	2.5	2.5	[32, 33, 35–39]
*r*_*N*_ *(mV*^*-1*^*)*	slope of the neuronal sigmoid function	0.45 or 0.56	0.56	[32, 33, 35–39]
*s*_*N*_ *(mV)*	mean firing threshold of the neuronal sigmoid function	6	6	[32, 33, 35–39]
*C*_*PC→IN*_	average number of synaptic contacts in the excitatory feedback loop	0.05 / 135 / 450	135	[32, 33, 35–39]
*C*_*PC→PC*_	average number of synaptic contacts in the excitatory feedback loop	0.05 / 108 / 240	13.5	[32, 33, 35–39]
*C*_*IN→IN*_	average number of synaptic contacts in the inhibitory feedback loop	0.08 / 33.75 / 400	81	[32, 33, 35–39]
*C*_*IN→PC*_	average number of synaptic contacts in the inhibitory feedback loop	0.06 / 33.75 / 280	13.5	[32, 33, 35–39]
*W (μM*.*s*^*-1*^*)*	gain coefficient of the glutamate release transfer function	-	18.46 (*)	[40]
*w*_*1*_ *(s*^*-1*^*)*	parameter of the glutamate release transfer function	-	90	[40]
*w*_*2*_ *(s*^*-1*^*)*	parameter of the glutamate release transfer function	-	33	[40]
*Z (μM*.*s*^*-1*^*)*	gain coefficient of the GABA release transfer function	-	613 (*)	[41]
*z*_*1*_ *(s*^*-1*^*)*	parameter of the GABA release transfer function	-	90	[41]
*z*_*2*_ *(s*^*-1*^*)*	parameter of the GABA release transfer function	-	33	[41]
*V*_*mg*_ *(μM*.*s*^*-1*^*)*	magnitude of the glutamate uptake sigmoid	-	5	[42–45]
*r*_*g*_ *(μM*^*-1*^*)*	slope of the glutamate uptake sigmoid	-	0.5	[42–45]
*s*_*g*_ *(μM)*	threshold of the glutamate uptake sigmoid	-	9	[42–45]
*V*_*m1*_ *(μM*.*s*^*-1*^*)*	Michaelis-Menten maximum velocity for GAT1 transporters (neurons)		5	[46–49]
*K*_*m1*_ *(μM)*	Michaelis-Menten concentration for GAT1 transporters (neurons)		24	[46–49]
*V*_*m3*_ *(μM*.*s*^*-1*^*)*	Michaelis-Menten maximum velocity for GAT3 transporters (astrocytes)		2	[46, 48–50]
*K*_*m3*_ *(μM)*	Michaelis-Menten concentration for GAT3 transporters (astrocytes)		8	[46, 48–50]
*V*_*gme*_ *(μM*.*s*^*-1*^*)*	rate of glutamate degradation in astrocytes	0.15 to 7.9	0.147 (*)	[51–53]
*V*_*gba*_ *(μM*.*s*^*-1*^*)*	rate of GABA degradation in astrocytes	-	1.984 (*)	[54]
*ε*_*n*_ *(s*^*-2*^*)*	efficacy of the neuronal contribution to the normalized flow dynamics	0.5 to 1	8 to 120	[3]
*ε*_*a*_ *(s*^*-2*^*)*	efficacy of the astrocytic contribution to the normalized flow dynamics	0.5 to 1	8 to 120	[3]
*τ*_*sn*_ *(s)*	time-constant for signal decay of the neuronal contribution to the normalized flow dynamics	0.4 to 0.8	0.4 to 1.9	[3]
*τ*_*sa*_ *(s)*	time-constant for signal decay of the astrocytic contribution to the normalized flow dynamics	0.4 to 0.8	0.4 to 1.9	[3]
*τ*_*fn*_ *(s*^*2*^*)*	time-constant for autoregulatory feedback of the neuronal contribution to the normalized flow dynamics	0.4 to 0.8	0.7 to 10.3	[3]
*τ*_*fa*_ *(s*^*2*^*)*	time-constant for autoregulatory feedback of the astrocytic contribution to the normalized flow dynamics	0.4 to 0.8	0.7 to 10.3	[3]

Parameter names are used throughout this study in all equations (S1 Table). The physiological description corresponds to the parameter meaning in the model. The typical value corresponds to the range of values taken from the papers listed in the reference column. Values of the neurotransmitter cycles were homogenized to the same unit by conversion, by considering that 1 *ml* of brain corresponds to 930 *mg* of tissue and 93 *mg* of proteins.

(*) Values obtained by stationary state (baseline) calculation (S3 File).

The average inhibitory post-synaptic potential *IPSP*_*PC*_ (from interneurons to pyramidal cells, Fig 2A) was similarly defined as:

$$\frac{d^{2}IPSP_{PC}}{dt^{2}}=BbC_{IN\to PC}FR_{IN}(t)-2b\frac{dIPSP_{PC}}{dt}-b^{2}IPSP_{PC}(t),$$ (5)

where *FR*_*IN*_ is the firing rate of interneurons, related to their *EPSP*_*IN*_ according to the equation:

$$FR_{IN}(t)=sigm\left(C_{IN\to IN}EPSP_{IN}(t),2e_{0},r_{N},s_{N}\right)$$ (6)

using the sigmoid function from Eq 4. The average excitatory post-synaptic potential *EPSP*_*IN*_ of interneurons depended on the firing rate *FR*_*PC*_ of pyramidal cells according to:

$$\frac{d^{2}EPSP_{IN}}{dt^{2}}=AaFR_{PC}(t)-2a\frac{dEPSP_{IN}}{dt}-a^{2}EPSP_{IN}(t),$$ (7)

where *FR*_*PC*_ was defined as the sigmoid function from Eq 4 applied to the simulated LFP, as follows:

$$FR_{PC}(t)=sigm\left(EPSP_{PC}(t)-IPSP_{PC}(t),2e_{0},r_{N},s_{N}\right).$$ (8)

### Comparison between the neural mass model and experimental interictal-like discharges

Simultaneous LFP-LD data were recorded after bicuculline injection and isolated events were extracted (Materials and Methods, S1 Fig). We found that the magnitude of the isolated discharges decreased over time, as bicuculline was washed out. For the nine extracted discharges, this magnitude varied (mean 6.38±1.33, *n* = 9) from *A*_*peak*_ = 8.14 *mV* (Fig 2B) to *A*_*peak*_ = 4.13 *mV* (Fig 2C). Parameters of the neural mass part (except for gains *G*_*i*_), described by Eqs 1–8, were set to values leading to simulated LFP as close as possible to the experimental discharges (Materials and Methods, S2 Fig). This was done by visualizing both the simulated and experimental LFP. Indeed, as parameters of the neural mass model are well known (see typical values and references in Table 1), we thus had a priori information about their effect on the temporal dynamics of the simulated epileptiform discharges. Note that the obtained parameters values (Table 1) were included in the typical range of the literature. Values of gains *G*_*i*_ in Eq 2 varied in order to obtain magnitudes of the simulated LFP reaching exactly the magnitudes *A*_*peak*_ of the extracted discharges. Due to bicuculline wash-out, the obtained values corresponded to reduced discharge magnitudes: *G*_*1*_ = 965, *G*_*2*_ = 929, *G*_*3*_ = 923, *G*_*4*_ = 810, *G*_*5*_ = 756, *G*_*6*_ = 690, *G*_*7*_ = 707, *G*_*8*_ = 673 and *G*_*9*_ = 535. Using these values, the simulated LFP (in black, Fig 2B and 2C) matched extremely well the LFP recording (in gray, Fig 2B and 2C). As the gain was the only modified parameter across simulated discharges, these results can be interpreted as the influence of bicuculline wash-out on a physiological state (here, the studied rat) depicted by the other pre-defined parameters. Besides, because the simulated discharges were the input to the remaining of the sequential model (S2 and S3 Figs), achieving a satisfactory agreement between the model and the data for the neuronal part of the model was essential in order to ensure a similar consistency for the subsequent compartments.

### A simple model of the neurotransmitter (glutamate and GABA) cycles

**Fig 3:**
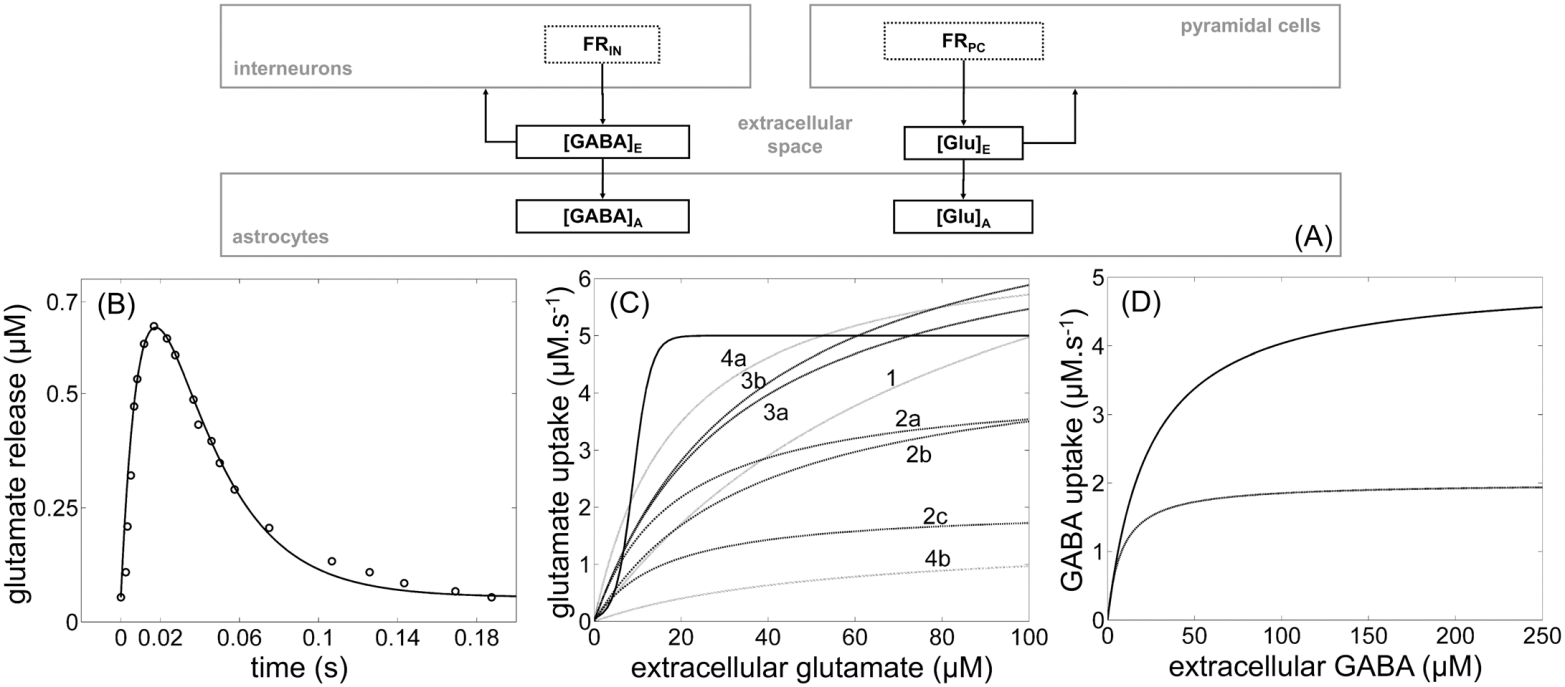
Modeling of the glutamate and GABA cycles according to the experimental literature. (A) Main physiological principles of glutamate and GABA cycles are glutamate and GABA releases by pyramidal cells and interneurons respectively, glutamate uptake by astrocytes and GABA uptake by both neurons and astrocytes (S1 File). (B) The glutamate release (solid line) from Eq 9 matches the experimental impulse response (circles) depicted in Fig 4 in [40] for the parameter set {*W* = 0.59, *w*_*1*_ = 90, *w*_*2*_ = 33}. (C) Comparison between the simulated glutamate uptake from Eq 11 and Michaelis-Menten representations {*V*_*M*_, *K*_*M*_} obtained from the experimental literature and converted to the same unit (Table 1). The Michaelis-Menten representations are numbered according to the experimental literature with 1: {*V*_*M*_ = 9.5, *K*_*M*_ = 91} for [42]; 2a: {*V*_*M*_ = 4.2, *K*_*M*_ = 18.6}, 2b: {*V*_*M*_ = 4.8, *K*_*M*_ = 37}, 2c: {*V*_*M*_ = 2, *K*_*M*_ = 16} for [44]; 4a: {*V*_*M*_ = 6.8, *K*_*M*_ = 18.9}, 4b: {*V*_*M*_ = 1.5, *K*_*M*_ = 54.9} for [45]. Setting parameters of Eq 11 to {*V*_*mg*_ = 5, *r*_*g*_ = 0.5, *s*_*g*_ = 9} led to a sigmoid function (solid line), which was close to the experimental measures for the usual physiological values of extracellular glutamate concentration (below 10 *μM*). (D) Comparison between Michaelis-Menten responses of the GABA uptake from Eqs 15 and 16 with {*V*_*m1*_ = 5, *K*_*m1*_ = 24} for GAT1 transport (neurons, in gray) and {*V*_*m1*_ = 2, *K*_*m1*_ = 8} for GAT3 transport (astrocytes, in black).

Neuro-glial interactions occurring at the glutamatergic and GABAergic synapses [55], i.e. between the neuronal and astrocytic compartments of the model, involve a variety of processes called neurotransmitter cycles. A first version of the neurotransmitter cycles [50, 56] was simplified (Fig 3A, S1 File) to glutamate and GABA releases and uptakes. When defining equations and parameter values, we considered the physiological interactions at the level of synapses and we converted them to the population scale according to the experimental literature (Table 1).

We started from Fig 4 from [40] giving the extracellular concentration of glutamate in response to a single action potential (impulse response). The shape of this response (Fig 3B), with very different rise time and decay time, led us to introduce the bi-exponential function described in [39, 57]. In order to adapt the model to the population scale, we applied this function to the firing rate *FR*_*PC*_ to define the glutamate release *Glu*_*N→E*_ as follows:

$$\frac{d^{2}Glu_{N\to E}}{dt^{2}}=Ww_{1}\text{exp}\left[w_{2}\frac{\text{ln}(w_{1}/w_{2})}{w_{1}-w_{2}}\right]FR_{PC}(t)-(w_{1}+w_{2})\frac{dGlu_{N\to E}}{dt}-w_{1}w_{2}Glu_{N\to E}(t).$$ (9)

The dynamics of GABA release by interneurons found in the experimental literature look like a bi-exponential function [58–60] with a variety of dynamic constants [41]. We thus chose the bi-exponential function [61] applied to the firing rate *FR*_*IN*_ for the GABA release *GABA*_*N→E*_ equation, as follows:

$$\frac{d^{2}GABA_{N\to E}}{dt^{2}}=Zz_{1}\text{exp}\left[z_{2}\frac{\text{ln}(z_{1}/z_{2})}{z_{1}-z_{2}}\right]FR_{IN}(t)-(z_{1}+z_{2})\frac{dGABA_{N\to E}}{dt}-z_{1}z_{2}GABA_{N\to E}(t).$$ (10)

Our NGV model considered glutamate uptake from both neurons and astrocytes (Fig 3A). Astrocytes demonstrate a sensing capacity to probe the extracellular space, i.e. glutamate uptake by astrocytes is activated when a given level of extracellular glutamate concentration is reached. Moreover, glutamate uptake by astrocytes presents a saturation effect. In order to take these mechanisms into account, we chose to represent the glutamate uptake *Glu*_*E→A*_ by astrocytes with the sigmoid function of Eq 4:

$$Glu_{E\to A}(t)=sigm(Glu_{E}(t),V_{mg},r_{g},s_{g}).$$ (11)

Glutamate reuptake by neurons being typically 10% [62] to 20% [63] of the total glutamate concentration taken from the extracellular space, we simply defined glutamate reuptake by neurons *Glu*_*E→N*_ as follows:

$$Glu_{E\to N}(t)=\frac{M}{1-M}Glu_{E}{{}_{\to }}_{A}(t)$$ (12)

where *M* corresponds to the fraction of glutamate reabsorbed by neurons. For simplification purposes, we set *M* = 0 in this study. The variation in the extracellular concentration of glutamate was defined as the difference between the release rate of Eq 9 and the uptake rate of Eqs 11 and 12, given by:

$$\frac{dGlu_{E}}{dt}=Glu_{N\to E}(t)-Glu_{E\to A}(t)-Glu_{E\to N}(t).$$ (13)

Complex mechanisms occur into the astrocytic compartment (S1 File). As a first approximation, we considered that the consumption rate *V*_*gme*_ of glutamate into the astrocytes was constant. This led to the astrocytic glutamate concentration *Glu*_*A*_ defined as follows:

$$\frac{dGlu_{A}}{dt}=Glu_{E\to A}(t)-V_{gme}.$$ (14)

As opposed to glutamate uptake, which is mainly achieved by astrocytes, GABA uptake is mainly due to reuptake by interneurons [64]. We kept the Michaelis-Menten representation (Table 1) used in the experimental literature to model GABA uptakes, whereas a sigmoid function was used to model glutamate uptake, which is a more significant process acting on CBF dynamics [50]. Actually, GABA transport and metabolism seem to involve less complex processes than those of glutamate [46, 65]. Therefore, the neuronal and astrocytic GABA uptakes were, respectively:

$$GABA_{E\to N}(t)=\frac{V_{m1}}{K_{m1}+GABA_{E}(t)}GABA_{E}(t)$$ (15)

and

$$GABA_{E\to A}(t)=\frac{V_{m3}}{K_{m3}+GABA_{E}(t)}GABA_{E}(t)$$ (16)

where *GABA*_*E*_ is the extracellular GABA concentration and {*V*_*m1*_, *K*_*m1*_, *V*_*m3*_, *K*_*m3*_} are the Michaelis-Menten parameters (Table 1). In this type of representation, the parameter *V*_*m*_ defines a saturation phenomenon (maximum rate) and the parameter *K*_*m*_ (Michaelis-Menten constant) defines the curve slope for increased values of the extracellular concentration. We described the variation of *GABA*_*E*_ by the difference between the release rate of Eq 10 and the uptake rates of Eqs 15 and 16, which led to the following equation:

$$\frac{dGABA_{E}}{dt}=GABA_{N\to E}(t)-GABA_{E\to A}(t)-GABA_{E\to N}(t).$$ (17)

In the same way as for glutamate degradation into the astrocytic compartment, a constant rate *V*_*gba*_ was considered for GABA degradation, so that the astrocytic GABA concentration was simply given by:

$$\frac{dGABA_{A}}{dt}=GABA_{E\to A}(t)-V_{gba}.$$ (18)

### Comparison between the modeled neurotransmitter cycles and the experimental literature

We did not have access to experimental data linked to these cycles, such as recordings with glutamate probes [66, 67]. The parameter values for the neurotransmitter cycle part of the model (Eqs 9–18) were thus chosen in the experimental range found in the experimental literature (Fig 3 and Table 1). For model-data comparison regarding glutamate release, we manually tuned parameters {*W*, *w*_*1*_, *w*_*2*_} of Eq 9 as close as possible (solid line in Fig 3B) to the experimental impulse response of an action potential (dotted line in Fig 3B) of [40]. In order to adapt the model from the synaptic scale of an action potential to the population scale of the firing rate *FR*_*PC*_, we kept the values of *w*_*1*_ and *w*_*2*_ that ensured physiological rise and decay times and we modified the magnitude *W* of the response according to the stationary state of the model (S3 File). The same methodology was followed to set the parameters {*Z*, *z*_*1*_, *z*_*2*_} of GABA release in Eq 10 (Table 1). We chose the sigmoid function of Eq 11 to represent glutamate uptake by astrocytes although experimental studies are usually based on Michaelis-Menten representation. As a consequence, we had to manually tune parameters {*V*_*mg*_, *r*_*g*_, *s*_*g*_} so that the resulting sigmoid was as close as possible to the different Michaelis-Menten experimental curves (Fig 3C). We thus adapted the parameters of the sigmoid in order to obtain, for typical values of *Glu*_*E*_ (range 0–10 *μM*) in our study, both the same level of saturation and the same slope as for the Michaelis-Menten curves. GABA uptake parameters {*V*_*m1*_, *K*_*m1*_} of the neuronal contribution of Eq 15 correspond to mGAT1 for mice and GAT1 for rats/humans (S1 File). Similarly, GABA uptake parameters {*V*_*m3*_, *K*_*m3*_} of the astrocytic contribution of Eq 16 correspond to mGAT4 for mice and GAT3 for rats/humans (S1 File). After unit conversion (Table 1), we chose the values {*V*_*m1*_ = 5, *K*_*m1*_ = 24, *V*_*m3*_ = 2, *K*_*m3*_ = 8} leading to a more important uptake for GAT1 than for GAT3 (Fig 3D) as depicted in the literature (Table 1, S1 File).

### Simulations of the neurotransmitter cycles agree with the phenomenological literature

**Fig 4:**
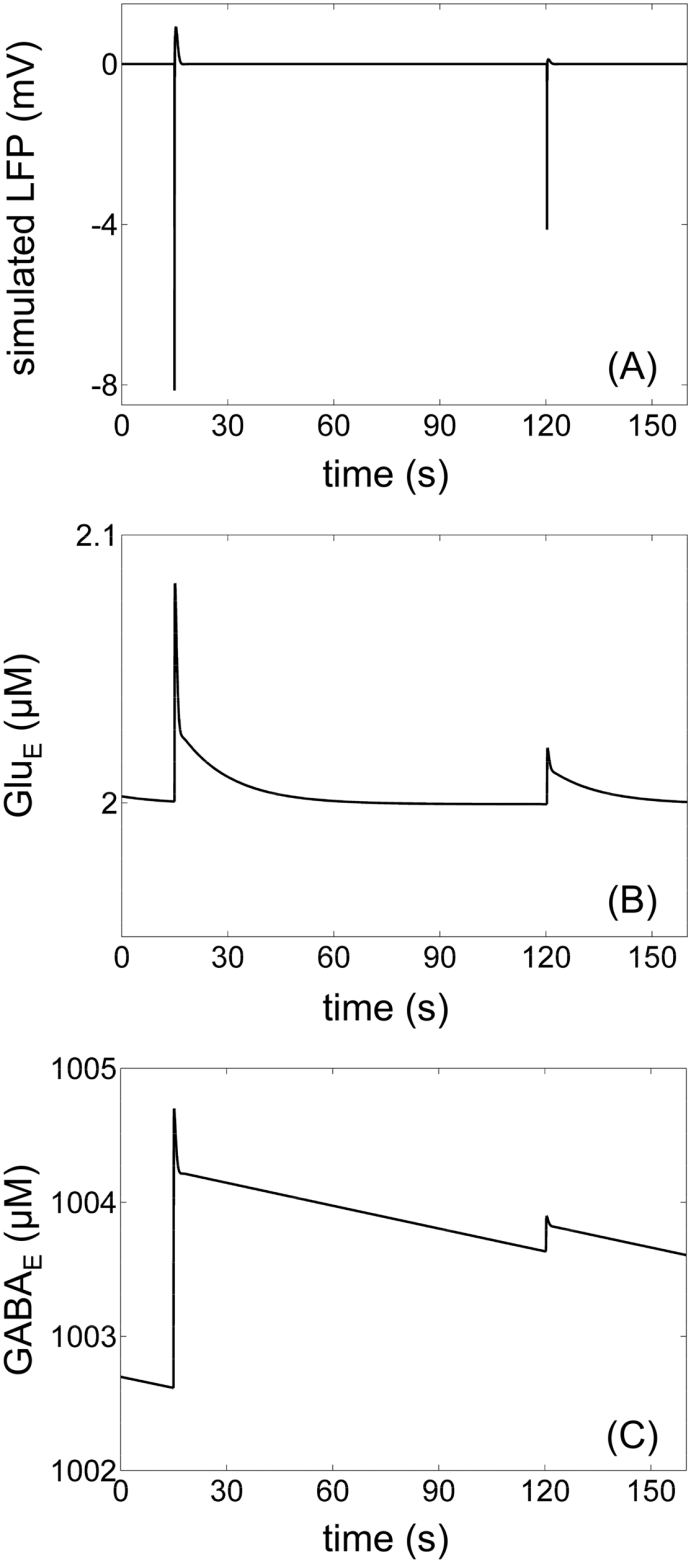
Temporal simulations of the NVG model. (A) Simulated LFP for discharges number 1 (with the highest level) and number 9 (with the lowest level) separated by 105 *s* reproduced bicuculline wash-out as a function of time. (B) Temporal simulation of the resulting extracellular concentration of glutamate *Glu*_*E*_ was in a good agreement with the temporal dynamics of experimental recordings with a glutamate probe such as those found in Fig 2 in [67]. (C) The resulting extracellular GABA concentration *GABA*_*E*_ had a slower dynamics than *Glu*_*E*_.

With pre-defined parameters for the neurotransmitter cycle part of the model, we were able to obtain the simulated temporal dynamics of the extracellular concentrations *Glu*_*E*_ and *GABA*_*E*_ (Fig 4B and 4C) for the highest and lowest level isolated discharges (Fig 4A) separated by 105 *s*.

The obtained dynamics of *Glu*_*E*_ (Fig 4B) were in good agreement with the experimental literature such as the dynamics depicted in Fig 2 in [67]. The dynamics of the extracellular GABA concentration *GABA*_*E*_ (Fig 4C) were more difficult to compare with the experimental literature because *GABA*_*E*_ is usually indirectly measured by inhibitory postsynaptic currents and there exists a wide variety of shapes and magnitdes of the responses [41]. The obtained *GABA*_*E*_ dynamics were slower than those of glutamate transport, which was consistent with the literature [68].

### A new representation of CBF changes: introducing both neuronal and astrocytic contributions

**Fig 5:**
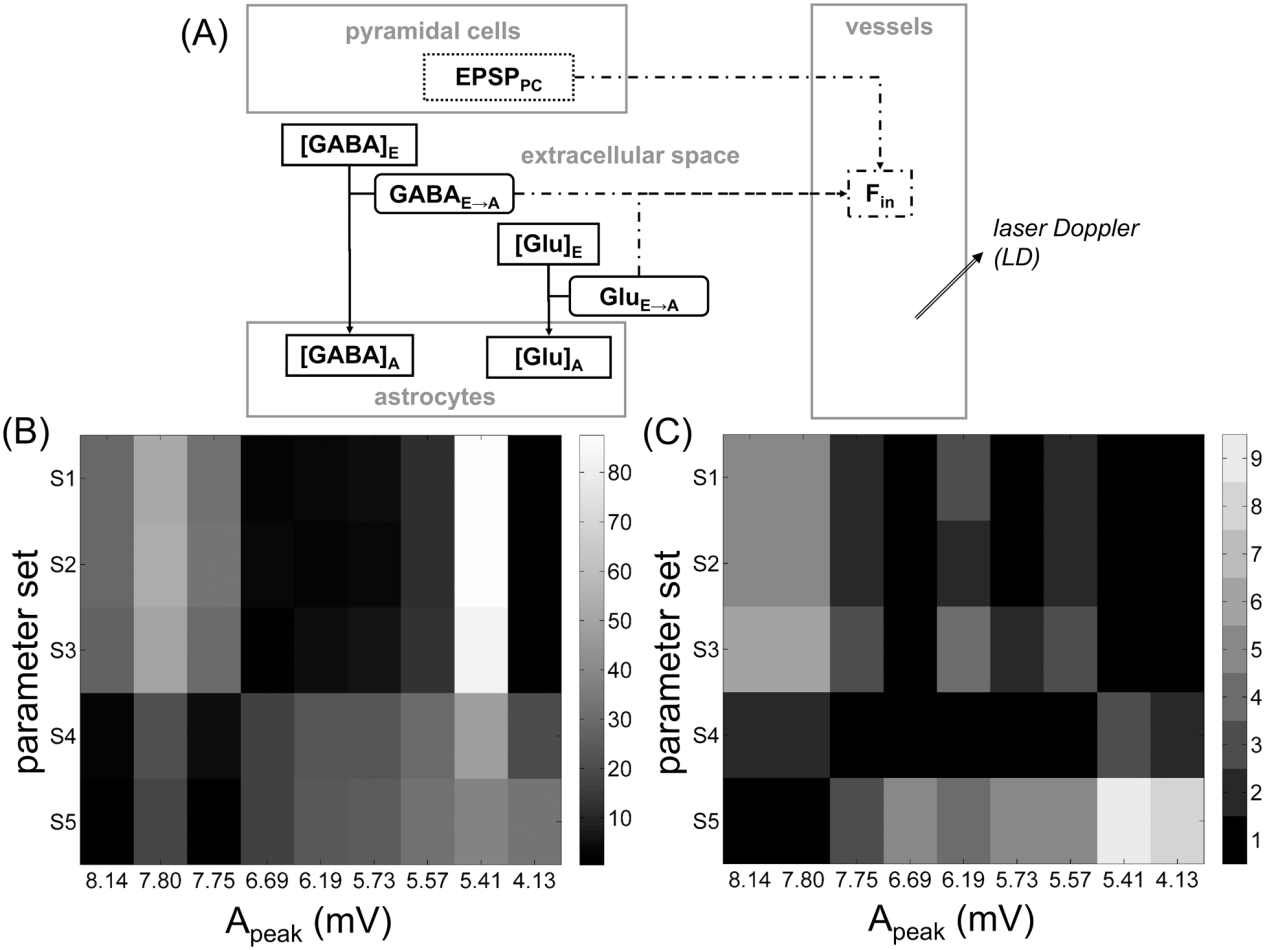
Modeling cerebral blood flow (CBF) dynamics. (A) CBF dynamics represented by the variable *F*_*in*_ consist of a neuronal contribution and an astrocytic contribution. (B) Model-data comparison was assessed by the relative error |*F*_*peak*,*simu*_—*F*_*peak*,*expe*_|/*F*_*peak*,*expe*_, where *F*_*peak*,*simu*_ is the CBF magnitude collected on the simulations and *F*_*peak*,*expe*_ is the CBF magnitude collected on the laser Doppler recording (Materials and Methods). This relative error (in %, coded in grayscale with black for lower values and white for higher values) was represented as a function of the magnitude *A*_*peak*_ of the extracted discharges and the parameter set leading to the magnitude *F*_*peak*,*simu*_. (C) Same as (B) for time *t*_*peak*_ of the main peak (Materials and Methods).

*Glu*_*E*_ and *GABA*_*E*_ uptakes by astrocytes contribute to a local increase in CBF (a phenomenon referred to as functional hyperemia) via a variety of vasoactive mediators (see [8, 69–71] for good reviews on the different mediators that could be involved). This increase is achieved through different mechanisms (S2 File). Interestingly, these mechanisms correspond to a slow indirect contribution of astrocytes and a rapid direct contribution of neurons [8, 72–74], represented in a parallel manner in our model (Fig 5A).

To our knowledge, only one experimental demonstration [7] has concluded that when isoflurane concentration varied from high to low, astrocytic calcium response (to visual stimuli) was reduced by 77±14%, compared with a 16±8% reduction for neuronal calcium response. Therefore, we considered that CBF dynamics, linked to calcium dynamics, could be approximately explained by a 80% contribution of astrocytes (*f*_*A*_*)* and a 20% contribution of neurons (*f*_*N*_*)*, which led to the equation:

$$f_{in}(t)=\frac{F_{in}(t)}{F_{0}}=0.8f_{A}(t)+0.2f_{N}(t),$$ (19)

where {*f*_*N*_, *f*_*A*_, *f*_*in*_} are variables normalized to the flow baseline *F*_*0*_, and *F*_*in*_ is the cerebral blood flow rate entering the voxel. In order to represent the neuronal contribution *f*_*N*_, corresponding to fast and direct increase by synaptic projections through dinoprostone (PGE2, cyclooxygenase (COX-2), and nitric oxyde (NO) (S2 File), we chose the well-known model of [3] described by the equation:

d2fNdt2=εn[EPSPPC(t)/normu1−1]−1τsndfNdt−fN(t)−1τfn, (20)

where *norm*_*u1*_ is the baseline value of *EPSP*_*PC*_. Likewise, we described the astrocytic contribution *f*_*A*_ to the flow dynamics, representing a slow activity via cascades of mediators (including glutamate and GABA) such as NO and epoxyeicosatrienoic acids (EETs), by the equation:

d2fAdt2=εa[(GluE→A(t)+GABAE→A(t))/normu2−1]−1τsadfAdt−fA(t)−1τfa, (21)

where *norm*_*u2*_ is the baseline value of the uptakes. In practice, *norm*_*u1*_ and *norm*_*u2*_ were computed on a slot of 30 *s* (initial value 1), setting the input characteristics to *σ*_*p*_ = 0 and *G*_*i*_ = 0 (see S1 Table for baseline values and S3 File for more details on the stationary state calculations). With these ultimate equations, the entire neuro-glio-vascular model (S3 Fig) is described by a system of ordinary differential equations (S1 Table).

### Model-data comparison between the CBF part of the model and the LD recording corresponding to the discharges

To explore the capacity of the model to represent epileptic phenomena, we decided to manually tune (S2 Fig) the resulting simulations of the total inflow *f*_*in*_ to reproduce the isolated laser Doppler recordings, as we assumed *f*_*in*_ to be directly related to these recordings (no observation equation). Isolated LD events were extracted together with the isolated discharges from the continuous (LFP-LD) dataset (Materials and Methods, S1 Fig). As the laser Doppler recording was very noisy, we first filtered the isolated events and defined their local baseline in the trough before the principal peak. This allowed us to compare the normalized variable *f*_*in*_ with the normalized isolated events. The difficulty to define a baseline value for the CBF led us to assume a standard shape for CBF events i.e. with neither initial dip nor post-stimulus undershoot. Actually, we were only interested in the magnitude of the main peak, not in the entire shape of the CBF dynamics, because we sought to study its relationship to the magnitude of the epileptiform discharges. We thus manually tuned the parameters {*ε*_*n*_, *τ*_*sn*_, *τ*_*fn*_, *ε*_*a*_, *τ*_*sa*_, *τ*_*fa*_} describing the neuronal and astrocytic flow contributions *f*_*N*_ and *f*_*A*_ of Eqs 20 and 21 in order to obtain a magnitude of *f*_*in*_ as close as possible to that of the isolated and filtered laser Doppler recordings. For the highest and lowest level discharges, we obtained five parameters sets (configurations) matching well the extracted LD recordings. Obtained sets of parameters were *S*_*1*_: {*ε*_*n*_ = 35, *τ*_*sn*_ = 1.3, *τ*_*fn*_ = 6.0, *ε*_*a*_ = 8, *τ*_*sa*_ = 1.6, *τ*_*fa*_ = 10.3}, *S*_*2*_: {*ε*_*n*_ = 35, *τ*_*sn*_ = 1.2, *τ*_*fn*_ = 5.8, *ε*_*a*_ = 31, *τ*_*sa*_ = 1.3, *τ*_*fa*_ = 3.0}, *S*_*3*_: {*ε*_*n*_ = 35, *τ*_*sn*_ = 1.2, *τ*_*fn*_ = 5.8, *ε*_*a*_ = 60, *τ*_*sa*_ = 0.8, *τ*_*fa*_ = 0.7}, *S*_*4*_:{*ε*_*n*_ = 22, *τ*_*sn*_ = 1.6, *τ*_*fn*_ = 10.3, *ε*_*a*_ = 44, *τ*_*sa*_ = 0.4, *τ*_*fa*_ = 0.7} and *S*_*5*_:{*ε*_*n*_ = 12, *τ*_*sn*_ = 1.0, *τ*_*fn*_ = 4.0, *ε*_*a*_ = 120, *τ*_*sa*_ = 1.9, *τ*_*fa*_ = 3.5}. Other configurations may lead to a good agreement between the simulations and the experimental flow events. Nevertheless, we did not analyze identification and uniqueness problems in the present study because as a first step, this model-data comparison was aimed at studying the capacity of the model to reproduce a single example of experimental data.

### The different sets of parameters contributing to CBF magnitude depend on the discharge magnitude

So as to explore quantitatively this model-data comparison, we collected (Materials and Methods, S1 Fig) the magnitude values *F*_*peak*,*simu*_ of the simulated variable *f*_*in*_ and compared them with the magnitude values *F*_*peak*,*expe*_ of the LD recordings, for each one of the nine events and for each one of the five parameter sets (Fig 5B). We found that the sets that best matched (i.e. the relative error was nearly zero) the experimental recordings were {*S*_*4*_, *S*_*5*_} for the highest level discharges and {*S*_*1*_, *S*_*2*_, *S*_*3*_} for the lowest level discharges. Likewise, we collected the instants of the flow peaks, called *t*_*peak*,*simu*_ for the simulated flow *f*_*in*_ and *t*_*peak*,*expe*_ for the LD recordings, respectively (Materials and Methods, S1 Fig). We also found a dependence between the adaptation of the parameter sets to *F*_*peak*,*expe*_ and the magnitude *A*_*peak*_ of the discharges (Fig 5C).

### Nonlinear relationship between CBF magnitude and epileptiform discharge magnitude in isolated events

Parameter sets correspond to the values of parameters {*ε*_*n*_, *τ*_*sn*_, *τ*_*fn*_, *ε*_*a*_, *τ*_*sa*_, *τ*_*fa*_} of Eqs 20 and 21 representing both the neuronal and astrocytic contributions *f*_*N*_ and *f*_*A*_ to the total inflow. In order to quantify the balance between the neuronal and astrocytic contributions to *f*_*in*_, without doing any specific study on these contributions, we defined an index *Q* based on the second-order differential equations describing *f*_*N*_ and *f*_*A*_ respectively (Materials and Methods). Depending on the parameter values, each set put more or less emphasis on either the neuronal contribution or the astrocytic contribution to *f*_*in*_, with *Q*>1 for emphasis on neuronal contribution and *Q* <1 for emphasis on astrocytic contribution, respectively. We obtained *Q* = {3.86, 2.56, 2.15, 0.46, 0.41} for the five sets of parameters {*S*_*1*_, *S*_*2*_, *S*_*3*_, *S*_*4*_, *S*_*5*_}, respectively. We therefore conclude that sets {*S*_*1*_, *S*_*2*_, *S*_*3*_} put more emphasis on neuronal contribution and that sets {*S*_*4*_, *S*_*5*_} put more emphasis on astrocytic contribution. Consequently, this indicates that neuronal configurations {*S*_*1*_, *S*_*2*_, *S*_*3*_} bore a close resemblance to the experimental LD recordings as the discharge magnitude *A*_*peak*_ was reduced. On the contrary, astrocytic configurations {*S*_*4*_, *S*_*5*_} seemed to better approximate the experimental recordings as *A*_*peak*_ increased. In physiological terms, it seems that for a low level discharge, the neuronal impact on the vessels is sufficient to elicit a flow response. However, when the discharge is high enough, astrocytic mechanisms are yet in action and lead to a more significant contribution of astrocytes to the flow. Interestingly, neuronal contribution was linear whereas astrocytic contribution was nonlinear (due to nonlinear uptake mechanisms). As a consequence, the relationship between neuronal activity (epileptiform discharges) and CBF dynamics (magnitude and timing of LD recordings) seems nonlinear for sufficiently high level discharges.

## Discussion

We proposed a new computational model developed at the scale of populations corresponding to multimodal acquisitions such as sEEG-fMRI. The model represents the forward signaling chain from neuronal activity to CBF changes, involving neurotransmitter (glutamate and GABA) cycles via the astrocytes. Particular efforts were done to achieve simple equations while describing the main physiological principles found in the literature.

We present a comparison of the model with bimodal (simultaneous LFP and laser Doppler) data acquired in the context of epilepsy. This sequential model-data comparison showed a good agreement of the model with the LFP recordings on isolated epileptiform discharges. This was obtained by manually tuning the neuronal parameters to patho-physiological values. We collected from the experimental literature a physiological range for the parameters of the glutamate and GABA cycles. Although we did not study in details the capacities and limitations of our model concerning these cycles, we showed its ability to reproduce typical dynamics of the extracellular concentrations of glutamate and GABA when elicited by neuronal events. We reproduced the laser Doppler recordings corresponding to the same isolated events with different sets of parameters. Although this comparison showed a limitation of our model in terms of identification of the whole CBF dynamics, it nevertheless provided mechanistic insights about the relationship between magnitudes of the neuronal activity and that of CBF. In particular, we showed that this relationship was due to neuronal contribution for low level neuronal events and to astrocytic contribution for higher level ones. Although it has been recently shown that the large majority of astrocytes responded with a calcium elevation to ictal but not interictal discharges [75], we found that an astrocytic contribution was already present when the interictal discharge was sufficiently important. Since astrocytic contribution to CBF increase is nonlinear, this result implies that the relationship between neuronal activity and CBF can be nonlinear, at least as far as sufficiently high level events are considered. A study of the balance between the dynamics of the neuronal and astrocytic contributions, respectively, by comparing our NGV model with data such as recordings in [7], would allow us to go further in the understanding of the sources of CBF changes.

More generally, understanding the neuro-glio-vascular coupling that exists between neurons, glial cells and vessels remains a difficult issue, given the number of metabolite interactions and the complexity of these interactions. Consequently, many studies may be considered starting from the existing model to understand other relationships, or expanding/modifying this model in order to take other mechanisms into account. For instance, we will be able to study the impact of discharge frequency on the nonlinear relationship between LFP and CBF, which could be directly linked to the extracellular concentration of neurotransmitters. More generally speaking, as the model includes a simple version of the glutamate and GABA cycles, we could further study the role of astrocytes in the excitation/inhibition balance. Indeed, this balance is important in a number of pathologies such as epilepsy. Under neuronal hyperactivity, apart from a massive release of neurotransmitters, a high level of metabolism activity (via a large potassium increase) is also induced in astrocytes. A longer-term perspective of this work is thus an extension of the model to metabolic mechanisms such as oxygen supply, which are also directly linked to astrocytic activity and CBF changes.

## Materials and Methods

All experimental protocols were approved by The Ethical Committee for Animal Experimentation of Marseille (approval number 30–03102012). The experimental protocol was performed in vivo on one Wistar-Han rat under general anesthesia (initially anesthetized with 5% and maintained under 2% isoflurane in 1 *l/min* of O_2_) delivered as a constant stream. The animal was euthanized by uretan injection during anesthesia.

### Simultaneous (LFP-LD) recordings in vivo

The animal was equipped with one tungsten electrode and one Doppler electrode, located above the somatosensory cortex. LFP were recorded by sharpened tungsten electrodes lowered to 500 *μm* into the cortex, close to the bicuculline injection site. CBF was recorded by a laser Doppler system (Perimed Periflux System 5000, Stockholm, Sweden; 0.03 *s* time constant, 780 *nm* laser). To measure CBF as locally as possible, we used a needle probe (Perimed probe 411) with a small separation (0.15 *mm*) between emitting and collecting light fibers [2]. To elicit epileptic discharges, bicuculline methochloride (2.5 *mM*, Abcam, UK) was infused at a rate of 200 *nl/min* during 5 *min* (1 *μl* total infusion) using a 5 *μl* microsyringe (Hamilton, 75RN neuro syringe) mounted to a micropump at a depth between 1000 and 1500 *μm* targeting the cortical layers III to VI. Epileptic discharges appeared about 7 *s* after the onset of the infusion.

### Extracted isolated events and chosen characteristics used for model-data comparison

The continuous (LFP-LD) data set is composed of isolated interictal-like discharges and burst discharges for LFP recordings, together with the simultaneous LD recordings corresponding to the CBF variations. We decided to compare the nonlinear model with the isolated events extracted from this continuous data set. To this end, we defined an isolated event as an interictal-like discharge on the LFP recording, i.e. an event which was sufficiently apart from the previous and following ones so that the corresponding LD variation had enough time to return to its (local) baseline.

Isolated discharges were extracted directly from the continuous dataset (no post-processing) and the magnitudes *A*_*peak*_ of their peak (S1 Fig) were collected manually. Note that the DC component of the LFP recording was hardware-filtered. LD recordings were first filtered with the Matlab^®^ (The Mathworks, Inc.) *eegfilt* function (1500 points) with a cutoff frequency of 0.5 *Hz* in order to obtain a smoothed version of the CBF temporal dynamics (S1 Fig). The CBF characteristics, collected from this smoothed version of the LD recording, were its magnitude *F*_*peak*_ and its duration *F*_*long*_ relative to the baseline (about 3 *mV*). We obtained nine isolated events with *F*_*long*_ varying from 22.2 *s* to 36.4 *s*, which were typical durations for the CBF to return to its baseline. We observed decreasing values of *A*_*peak*_ as the bicuculline local concentration was washed out over time.

### A simple but physiologically-relevant model

We proceeded by incorporating a number of intermediate variables and the main pathways involved in NGV coupling, from the physiological literature [46]. In order to reduce the subsequent identification problems and the global model complexity in terms of the resulting number of differential equations, a refinement work was done at the same time for both the selection of physiological variables and the selection of the pathways to be taken into account, from the cellular to the mesoscopic level. Likewise, we defined mathematically each relationship with the objective to obtain equations as simple as possible, while keeping their physiological meaning. If appropriate equations existed in the literature, then they were used directly. Otherwise, we adapted existing equations or defined new ones.

### Methodology for model-data comparison

The goal of model-data comparison was to study the capacity of the model to reproduce the extracted isolated events. The objective was to find at least one set of parameter values leading to simulated variables with a magnitude as close as possible to that of the corresponding recordings. The simulated variables LFP and *f*_*in*_ are located at the extreme sides of the (neural mass, glutamate and GABA cycles and sum) chain that constitutes the model (S2 Fig). In practice, we took advantage of the forward property of this chain, from neuronal activity to CBF changes [76], to conduct the comparison in a sequential manner (Materials and Methods and S2 Fig) from parameters of the input noise *p* of Eqs 1 and 2 to parameters of the neuronal and astrocytic contributions of Eqs 20 and 21. We chose the parameter values of the model in order to reproduce on the one hand, the dynamics observed in the simultaneous data for the observed (neuronal and vascular) parts of the model; on the other hand, the experimental literature for the intermediate non-observed (neurotransmitters cycles) part of the model.

### Definition of ratio Q

We defined the ratio *Q* according to the following considerations. The neuronal and astrocytic contributions to the total inflow *f*_*in*_, *f*_*N*_, and *f*_*A*_ respectively, are given by the equations:

{d2fNdt2=εnu1(t)−1τsndfNdt−fN(t)−1τfnd2fAdt2=εau2(t)−1τsadfAdt−fA(t)−1τfa,

where *u*_*1*_ and *u*_*2*_ are the inputs defined in Eqs 20 and 21. For these second-order systems, the magnitudes of the impulse responses are directly linked to the efficacy parameters *ε*_*n*_ and *ε*_*a*_. Likewise, the durations of their responses are related to the quantities *τ*_*fn*_/*τ*_*sn*_^2^ and *τ*_*fa*_/*τ*_*sa*_^2^. Therefore, the quantities *S*_*neu*_ = *ε*_*n*_.*τ*_*fn*_/*τ*_*sn*_^2^ and *S*_*ast*_ = *ε*_*a*_. *τ*_*fa*_/*τ*_*sa*_^2^ describe quantitatively the importance of the responses *f*_*N*_ and *f*_*A*_ on *f*_*in*_, respectively. The ratio *Q* = *S*_*neu*_/*S*_*ast*_ thus gives an idea of the impact of the neuronal contribution *f*_*N*_ on the total inflow *f*_*in*_, compared with the astrocytic contribution *f*_*A*_.

## Supporting Information

S1 Dataset Simultaneously recorded (LFP-LD) isolated events used in this study. For each extracted event *i*, time samples are denoted by *ti*, LFP samples by *lfpi* and laser Doppler samples by *fi*.
(MAT)

S1 Fig Example of an isolated event extracted from the simultaneous (LFP-LD) recordings. (A) The chosen characteristic collected on the LFP recording (in gray) was its peak magnitude *A*_*peak*_ from baseline (dashed line). (B) The chosen characteristics collected on a smoothed version (in black, see Materials and Methods) of the direct LD recording (in gray) were its peak magnitude *F*_*peak*_ from the (local) baseline, its duration *F*_*long*_, and the time *t*_*peak*_ of the peak.
(TIFF)

S2 Fig Model-data comparison between the forward NGV model and isolated events from simultaneous (LFP-LD) recordings. Isolated discharges were used to manually tune the neural mass part of the model and the input *p*; the experimental literature was used to set the parameters of the glutamate and GABA cycles with physiologically-relevant values; the smoothed versions (in black) of the isolated LD data (in gray) corresponding to the isolated discharges were used to manually tune the CBF part of the model leading to the output *f*_*in*_. The symbol Σ corresponds to the sum of Eq 19.
(TIFF)

S3 Fig Proposed neuro-glio-vascular (NGV) model and its link to multimodal recordings. Dynamical variables are encapsulated in squares (S1 Table). The model input is a noise *p* representing the influence of the environment (average density of afferent action potentials and bicuculline injection). Electrophysiological relationships (dotted lines) between the pyramidal cells compartment and the interneurons compartment lead to the neuronal activity measured by local field potential (LFP). Glutamate and GABA neurotransmitters are released in the extracellular space and recycled (solid lines) by both neuronal and astrocytes compartments. These activities lead to the cerebral blood flow (CBF) dynamics (dotted-dashed lines) represented in the vascular compartment and measured by laser Doppler (LD).
(TIFF)

S1 File Physiological literature leads to a simple version of the glutamate and GABA cycles. (DOC)

S2 File Neuronal and astrocytic contributions to CBF changes explained by the physiological literature. (DOC)

S3 File Stationary state calculations and deduced parameters. (DOC)

S1 Table Ordinary differential equations (ODE) describing the proposed neuro-glio-vascular model. State variables are specified, together with their initial value (stationary state). Parameter description and values are given in S1 Table. (°) Chosen from [54]. (°°) Chosen to be the average in the range 2070–2630 *μM* mentioned in [77]. (*) Values obtained by stationary state (baseline) calculation (S3 File). Input is *p(t)* of Eqs 1 and 2. The integration of this ODE system by numerical methods such as Runge-Kutta 4 leads to the simulation of the output of the model, the total cerebral blood inflow given by *f*_*in*_ *(t)* = 0.8*f*_*A*_*(t)*+0.2*f*_*N*_*(t)*.
(DOC)
