# Supplementary figures and images for: A New Computational Model for Neuro-Glio-Vascular Coupling: Astrocyte Activation Can Explain Cerebral Blood Flow Nonlinear Response to Interictal Events
Source: PLoS One. 2016 Feb 5;11(2):e0147292. doi: 10.1371/journal.pone.0147292 (PMC4743967; doi:10.1371/journal.pone.0147292)

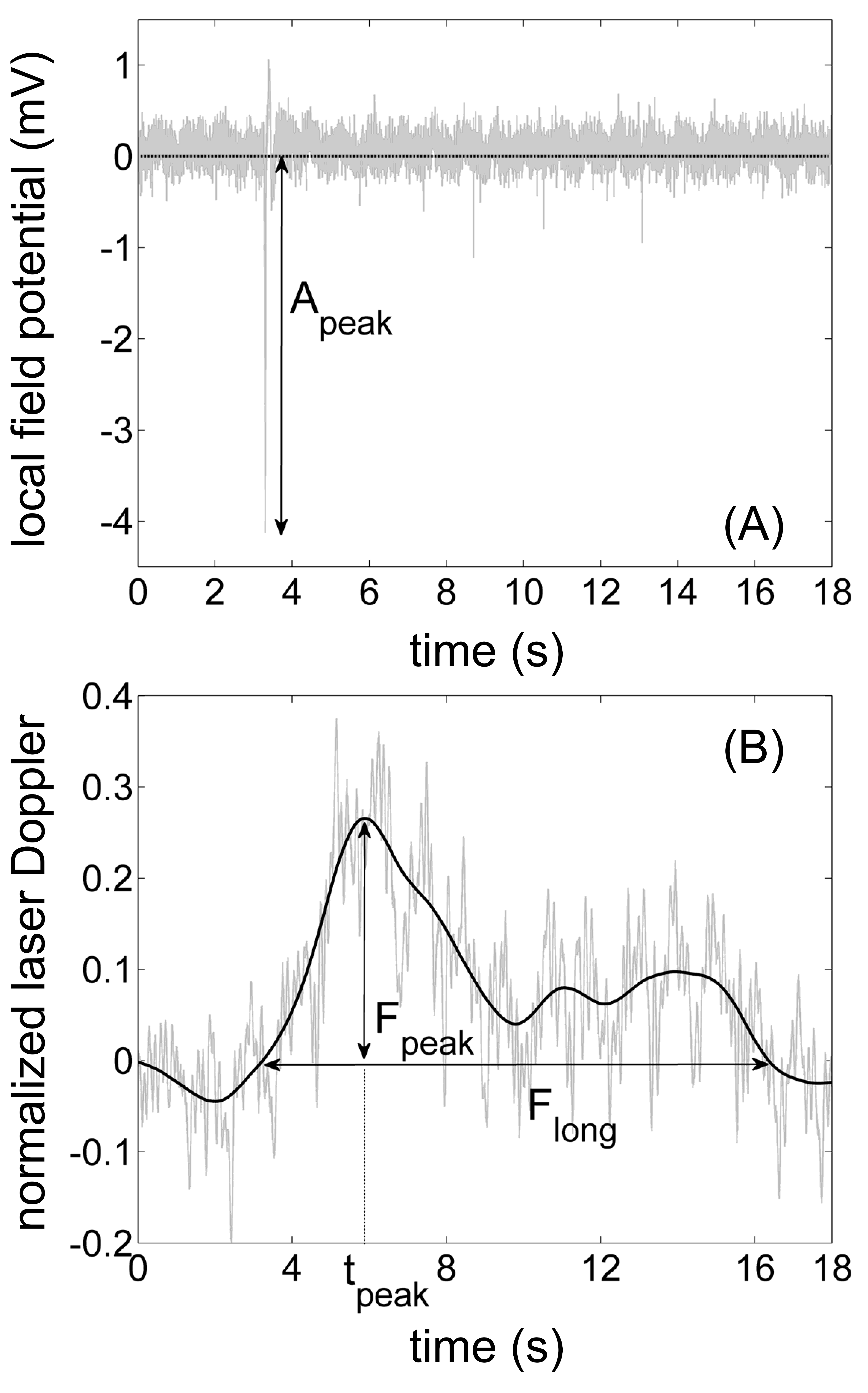

Supplement: S1 Fig — (A) The chosen characteristic collected on the LFP recording (in gray) was its peak magnitude Apeak from baseline (dashed line). (B) The chosen characteristics collected on a smoothed version (in black, see Materials and Methods) of the direct LD recording (in gray) were its peak magnitude Fpeak from the (local) baseline, its duration Flong, and the time tpeak of the peak. (TIFF) [file pone.0147292.s002.tiff]

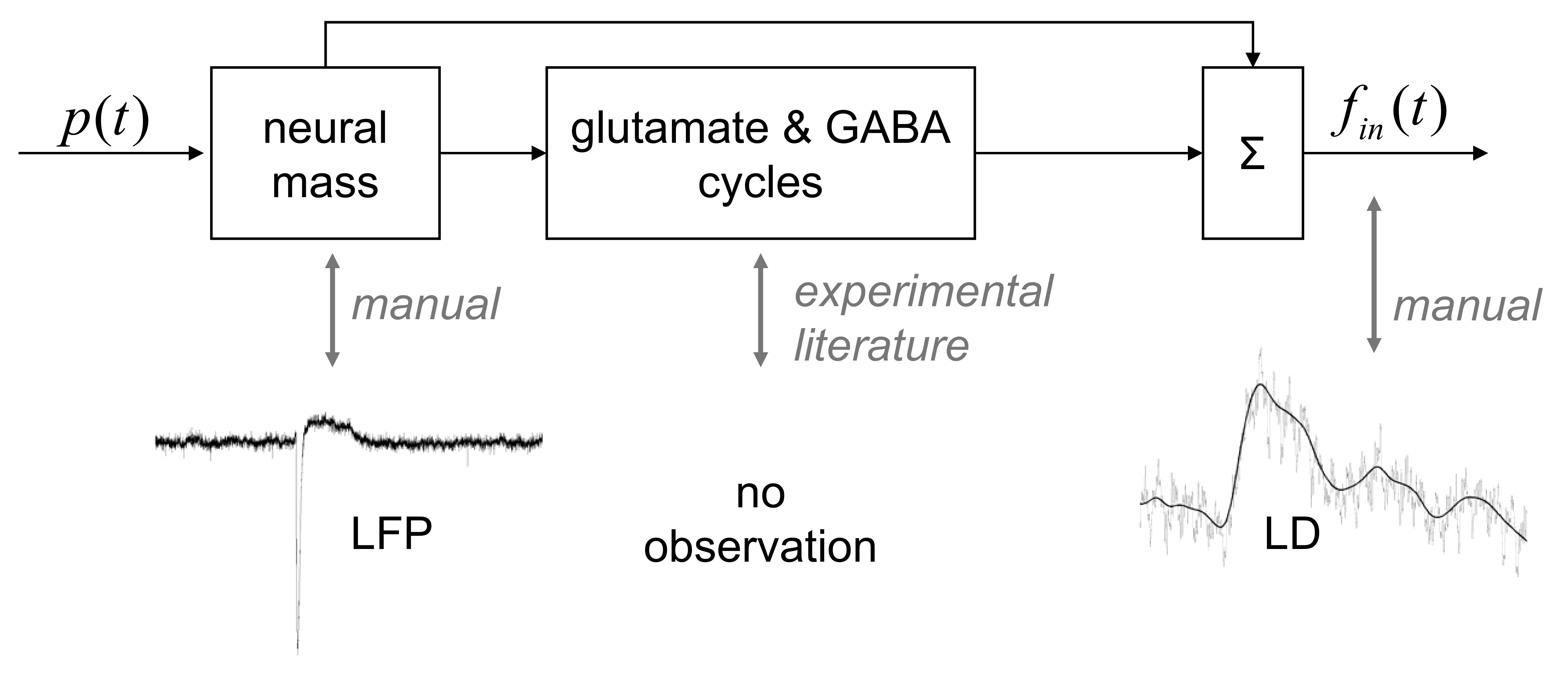

Supplement: S2 Fig — Isolated discharges were used to manually tune the neural mass part of the model and the input p; the experimental literature was used to set the parameters of the glutamate and GABA cycles with physiologically-relevant values; the smoothed versions (in black) of the isolated LD data (in gray) corresponding to the isolated discharges were used to manually tune the CBF part of the model leading to the output fin. The symbol Σ corresponds to the sum of Eq 19. (TIFF) [file pone.0147292.s003.tiff]

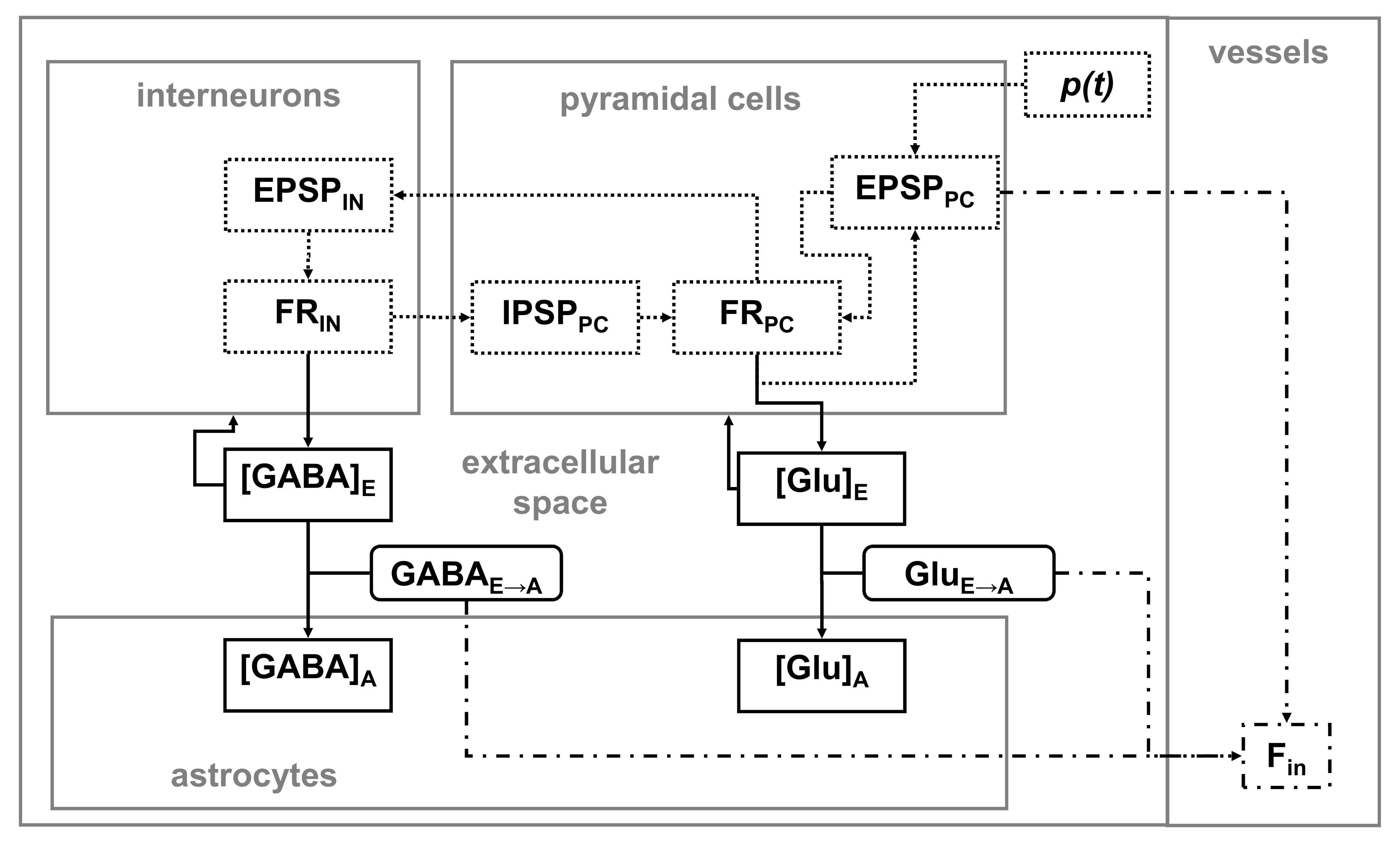

Supplement: S3 Fig — Dynamical variables are encapsulated in squares (S1 Table). The model input is a noise p representing the influence of the environment (average density of afferent action potentials and bicuculline injection). Electrophysiological relationships (dotted lines) between the pyramidal cells compartment and the interneurons compartment lead to the neuronal activity measured by local field potential (LFP). Glutamate and GABA neurotransmitters are released in the extracellular space and recycled (solid lines) by both neuronal and astrocytes compartments. These activities lead to the cerebral blood flow (CBF) dynamics (dotted-dashed lines) represented in the vascular compartment and measured by laser Doppler (LD). (TIFF) [file pone.0147292.s004.tiff]
