# Supplementary material for: A New Computational Model for Neuro-Glio-Vascular Coupling: Astrocyte Activation Can Explain Cerebral Blood Flow Nonlinear Response to Interictal Events
Source: PLoS One. 2016 Feb 5;11(2):e0147292. doi: 10.1371/journal.pone.0147292 (PMC4743967; doi:10.1371/journal.pone.0147292)
Supplement: S1 File — (DOC) [file pone.0147292.s005.doc]

**S1 File. Physiological literature leads to a simple version of the glutamate and GABA cycles.**

During neuronal activity, glutamate release by the presynaptic compartment is uptook by astrocytes and engaged into the glutamate-glutamine cycle . Glutamate uptake by astrocytes and its consumption into the glutamine cycle is the major pathway after glutamate is released in the extracellular space. The contribution of GABA to neuro-glial interactions is almost as important as glutamate contribution due to the multiple mechanisms (including GABA uptake) involved by inhibitory GABAergic interneurons . The contribution of GABA to the glutamate-glutamine cycle is more significant than its involvement in the citric acid cycle . Also, GABA consumption through this type of “intern” cycles (GABA shunt and TCA cycle) is considered as a secondary mechanism compared with the GABA uptake by interneurons . Consequently, consumptions of glutamate and GABA through the TCA cycles (neuronal and astrocytic) were simply considered as the constant velocities $V_{gme}$ and $V_{gba}$ (see S1 Table). Although there is an interesting link between glutamine and metabolic coupling , it was not necessary to include this variable in order to achieve a coupling between neuronal activity and CBF because the metabolic relationships around glucose/oxygen consumption were not represented in our model (neither glycogen considerations). Moreover, as a first step of the modeling process, we did not take into account possible glutamate transport from brain to vessels . Finally, the remaining considerations about neurotransmitter cycles are only the glutamate and GABA releases and uptakes (solid lines in S3 Fig.). Glutamate uptake by neurons is an active mechanism by EAAT1-5 transporters . Glutamate uptake by astrocytes is also an active mechanism , mainly by the glutamate transporters (GLAST,GLT-1) in rats and (EAAT1,EAAT2) in humans . GABA uptake by both neurons and astrocytes is due to active mechanisms via GABA transporters. The HUGO nomenclature gives a description of the GABA transporters of interest in our study , which are mGAT1 (for mice) and GAT1 (for rats and humans) for interneurons reuptake; mGAT4 (for mice) and GAT3 (for rats and humans) for astrocytic uptake. We used these considerations to extract the corresponding parameters values from the experimental literature (see S1 Table).

1. Lebon V, Petersen KF, Cline GW, Shen J, Mason GF, Dufour S, et al. Astroglial contribution to brain energy metabolism in humans revealed by 13C nuclear magnetic resonance spectroscopy: elucidation of the dominant pathway for neurotransmitter glutamate repletion and measurement of astrocytic oxidative metabolism. The Journal of neuroscience : the official journal of the Society for Neuroscience. 2002;22(5):1523-31.

2. Anderson CM, Swanson RA. Astrocyte glutamate transport: review of properties, regulation, and physiological functions. Glia. 2000;32(1):1-14.

3. Buzsaki G, Kaila K, Raichle M. Inhibition and brain work. Neuron. 2007;56(5):771-83.

4. Patel AB, de Graaf RA, Mason GF, Rothman DL, Shulman RG, Behar KL. The contribution of GABA to glutamate/glutamine cycling and energy metabolism in the rat cortex in vivo. Proc Natl Acad Sci U S A. 2005;102(15):5588-93.

5. Liang SL, Carlson GC, Coulter DA. Dynamic regulation of synaptic GABA release by the glutamate-glutamine cycle in hippocampal area CA1. The Journal of neuroscience : the official journal of the Society for Neuroscience. 2006;26(33):8537-48.

6. Rothman DL, De Feyter HM, de Graaf RA, Mason GF, Behar KL. 13C MRS studies of neuroenergetics and neurotransmitter cycling in humans. NMR in biomedicine. 2011;24(8):943-57.

7. Helms HC, Madelung R, Waagepetersen HS, Nielsen CU, Brodin B. In vitro evidence for the brain glutamate efflux hypothesis: brain endothelial cells cocultured with astrocytes display a polarized brain-to-blood transport of glutamate. Glia. 2012;60(6):882-93.

8. Danbolt NC. Glutamate uptake. Progress in neurobiology. 2001;65(1):1-105.

9. Shigeri Y, Seal RP, Shimamoto K. Molecular pharmacology of glutamate transporters, EAATs and VGLUTs. Brain research Brain research reviews. 2004;45(3):250-65.

10. Sheldon AL, Robinson MB. The role of glutamate transporters in neurodegenerative diseases and potential opportunities for intervention. Neurochemistry international. 2007;51(6-7):333-55.

11. Schousboe A, Bak LK, Waagepetersen HS. Astrocytic Control of Biosynthesis and Turnover of the Neurotransmitters Glutamate and GABA. Frontiers in endocrinology. 2013;4:102.

12. Kim K, Lee SG, Kegelman TP, Su ZZ, Das SK, Dash R, et al. Role of excitatory amino acid transporter-2 (EAAT2) and glutamate in neurodegeneration: opportunities for developing novel therapeutics. Journal of cellular physiology. 2011;226(10):2484-93.

13. Conti F, Minelli A, Melone M. GABA transporters in the mammalian cerebral cortex: localization, development and pathological implications. Brain research Brain research reviews. 2004;45(3):196-212.

14. Rowley NM, Madsen KK, Schousboe A, Steve White H. Glutamate and GABA synthesis, release, transport and metabolism as targets for seizure control. Neurochemistry international. 2012;61(4):546-58.
