# Supplementary material for: A New Computational Model for Neuro-Glio-Vascular Coupling: Astrocyte Activation Can Explain Cerebral Blood Flow Nonlinear Response to Interictal Events
Source: PLoS One. 2016 Feb 5;11(2):e0147292. doi: 10.1371/journal.pone.0147292 (PMC4743967; doi:10.1371/journal.pone.0147292)
Supplement: S2 File — (DOC) [file pone.0147292.s006.doc]

**S2 File. Neuronal and astrocytic contributions to CBF changes explained by the physiological literature.**

A variety of vessels cells (endothelial cells, smooth muscle cells and pericytes) allows the conversion from chemical vasoactive mediators to a mechanical action (local variation of the cerebral blood volume) leading to CBF increase. In the past decades, different pathways have been hypothesized to participate in CBF changes (increase and regulation). The lack of energy or oxygen (also known as reactive hyperemia) proved not to be a candidate for flow increase , so that we did not include this hypothesis into the model. The stretch of astrocyte endfeet on vessels was at the origin of studies on the impact of astrocytes on flow increase. Such studies conclude that astrocytes trigger local vasodilation when activated by glutamate . This astrocytic signaling is done by the intermediate of a number of vasoactive mediators such as epoxyeicosatrienoic acids (EETs) and hydroxyicosatetraenoic acids (HETEs).

In parallel, evidence for a neuronal signaling pathway was observed confronting BOLD or CBF dynamics with neuronal activity (LFP and multi-unit activity). A certain number of articles showed that BOLD and/or CBF better correlate with post-synaptic potential recorded by LFP rather than with firing rates recorded by MUA . These neuronal activities lead to the main mediators (GABA, NO for interneurons and (PGE2, COX-2 for pyramidal cells). We decided to reflect this pathway through a neuronal contribution to the CBF. As post-synaptic potentials and firing rates are correlated (with an integration function, see Eqs. (5)-(8) of the neural mass model), we considered that a neuronal contribution also has an impact on CBF via pyramidal cells , the contribution by vasoactive mediators being necessary but not sufficient . Although we chose to increase the CBF by the variable $EPSP_{PC}$, the influence of excitation/inhibition balance on CBF dynamics is (indirectly) taken into account because inhibition is included via the GABA cycle originating from $EPSP_{IN}$ of the neuronal activity loop.

1. Iadecola C. Neurovascular regulation in the normal brain and in Alzheimer's disease. Nature reviews Neuroscience. 2004;5(5):347-60.

2. Iadecola C, Nedergaard M. Glial regulation of the cerebral microvasculature. Nature neuroscience. 2007;10(11):1369-76.

3. Girouard H, Iadecola C. Neurovascular coupling in the normal brain and in hypertension, stroke, and Alzheimer disease. Journal of applied physiology. 2006;100(1):328-35.

4. Raichle ME, Mintun MA. Brain work and brain imaging. Annu Rev Neurosci. 2006;29:449-76.

5. Attwell D, Iadecola C. The neural basis of functional brain imaging signals. Trends in neurosciences. 2002;25(12):621-5.

6. Lin AL, Gao JH, Duong TQ, Fox PT. Functional neuroimaging: a physiological perspective. Frontiers in neuroenergetics. 2010;2.

7. Zonta M, Angulo MC, Gobbo S, Rosengarten B, Hossmann KA, Pozzan T, et al. Neuron-to-astrocyte signaling is central to the dynamic control of brain microcirculation. Nature neuroscience. 2003;6(1):43-50.

8. Cauli B, Hamel E. Revisiting the role of neurons in neurovascular coupling. Frontiers in neuroenergetics. 2010;2:9.

9. Mathiesen C, Caesar K, Akgoren N, Lauritzen M. Modification of activity-dependent increases of cerebral blood flow by excitatory synaptic activity and spikes in rat cerebellar cortex. The Journal of physiology. 1998;512 ( Pt 2):555-66.

10. Logothetis NK, Wandell BA. Interpreting the BOLD signal. Annual review of physiology. 2004;66:735-69.

11. Logothetis NK. What we can do and what we cannot do with fMRI. Nature. 2008;453(7197):869-78.

12. Figley CR, Stroman PW. The role(s) of astrocytes and astrocyte activity in neurometabolism, neurovascular coupling, and the production of functional neuroimaging signals. Eur J Neurosci. 2011;33(4):577-88.

13. Rauch A, Rainer G, Logothetis NK. The effect of a serotonin-induced dissociation between spiking and perisynaptic activity on BOLD functional MRI. Proc Natl Acad Sci U S A. 2008;105(18):6759-64.

14. Niessing J, Ebisch B, Schmidt KE, Niessing M, Singer W, Galuske RA. Hemodynamic signals correlate tightly with synchronized gamma oscillations. Science. 2005;309(5736):948-51.

15. Lippert MT, Steudel T, Ohl F, Logothetis NK, Kayser C. Coupling of neural activity and fMRI-BOLD in the motion area MT. Magnetic resonance imaging. 2010;28(8):1087-94.

16. Viswanathan A, Freeman RD. Neurometabolic coupling in cerebral cortex reflects synaptic more than spiking activity. Nature neuroscience. 2007;10(10):1308-12.

17. Mukamel R, Gelbard H, Arieli A, Hasson U, Fried I, Malach R. Coupling between neuronal firing, field potentials, and FMRI in human auditory cortex. Science. 2005;309(5736):951-4.

18. Lauritzen M. Reading vascular changes in brain imaging: is dendritic calcium the key? Nature reviews Neuroscience. 2005;6(1):77-85.

19. Tagamets MA, Horwitz B. Interpreting PET and fMRI measures of functional neural activity: the effects of synaptic inhibition on cortical activation in human imaging studies. Brain research bulletin. 2001;54(3):267-73.
