# Supplementary material for: A New Computational Model for Neuro-Glio-Vascular Coupling: Astrocyte Activation Can Explain Cerebral Blood Flow Nonlinear Response to Interictal Events
Source: PLoS One. 2016 Feb 5;11(2):e0147292. doi: 10.1371/journal.pone.0147292 (PMC4743967; doi:10.1371/journal.pone.0147292)
Supplement: S3 File — (DOC) [file pone.0147292.s007.doc]

**S3 File. Stationary state calculations and deduced parameters.**

We detail here the calculations of the stationary state for the ODE system representing our neuro-glio-vascular model (S1 Table). For the neuronal part of the model, this work was done in and particularly linking the mean $m_{B}$ of the input to the baseline $\left.LFP\right|_{b}$ of the LFP through the equation:

$\begin{matrix} \left.LFP\right|_{b}=\frac{A}{a}m_{B}+\frac{A}{a}C_{PC\to PC}sigm\left(\frac{A}{a}C_{PC\to IN}sigm\left(\left.LFP\right|_{b},2e_{0},r_{N},s_{N}\right)\right) \\ -\frac{B}{b}C_{IN\to PC}sigm\left(\frac{A}{a}C_{IN\to IN}sigm\left(\left.LFP\right|_{b},2e_{0},r_{N},s_{N}\right)\right) \end{matrix}$ **(1)**

which can be solved by *fsolve* Matlab function and leading to $\left.LFP\right|_{b}=0.25mV$ for parameters of Table 1 of the manuscript. From this baseline value, one has access to

$\left.FR_{PC}\right|_{b}=sigm\left(\left.LFP\right|_{b},2e_{0},r_{N},s_{N}\right)$ **(2)**

and thus, considering $\frac{d^{2}EPSP_{IN}}{dt^{2}}=\frac{dEPSP_{IN}}{dt}=0$ in Eq. (6) of the manuscript,

$\left.EPSP_{IN}\right|_{b}=\left.\frac{A}{a}FR_{PC}\right|_{b}.$ **(3)**

With this expression, one can deduce the stationary state of the electrophysiological variables of the model through the system:

$\left\{\begin{matrix} \left.FR_{IN}\right|_{b}=sigm\left(\left.C_{IN\to IN}EPSP_{IN}\right|_{b},2e_{0},r_{N},s_{N}\right) \\ \left.FR_{back}\right|_{b}=sigm\left(\left.C_{PC\to IN}EPSP_{IN}\right|_{b},2e_{0},r_{N},s_{N}\right) \\ \left.EPSP_{PC}\right|_{b}=\frac{A}{a}m_{B}+\frac{A}{a}C_{PC\to PC}sigm\left(\left.C_{PC\to IN}EPSP_{IN}\right|_{b},2e_{0},r_{N},s_{N}\right) \end{matrix}\right..$ **(4)**

Note that the normalization variable $norm_{u1}$ in the neuronal flow contribution of Eq. (19) of the manuscript is simply $norm_{u1}=\left.EPSP_{PC}\right|_{b}$. We obtained $\left.FR_{PC}\right|_{b}=0.147s^{-1}$ and $\left.EPSP_{IN}\right|_{b}=48\mu V$ with the parameters of the model (Table 1), which led to $\left.FR_{IN}\right|_{b}=0.207s^{-1}$, $\left.FR_{back}\right|_{b}=0.237s^{-1}$ and gave access to the baseline of the average inhibitory post-synaptic potential:

$\left.IPSP_{PC}\right|_{b}=\left.\frac{B}{b}C_{IN\to PC}FR_{IN}\right|_{b}.$ **(5)**

Numerical application with parameters of Table 1 led to the values $\left.EPSP_{PC}\right|_{b}=3.097mV$ and $\left.IPSP_{PC}\right|_{b}=3.348mV$. With the baseline value of the firing rate $\left.FR_{PC}\right|_{b}$, the stationary state of variables of Eq. (8) and Eq. (12) of the manuscript representing the glutamate cycle can be calculated by:

$\left\{\begin{matrix} \left.Glu_{N\to E}\right|_{b}=\frac{W}{w_{2}}c(w_{1},w_{2})\left.FR_{PC}\right|_{b} \\ \left.Glu_{E}\right|_{b}=s_{g}-\frac{1}{r_{g}}\ln\left(\frac{V_{mg}}{(1-M)\left.Glu_{N\to E}\right|_{b}}-1\right) \end{matrix}\right..$ **(6)**

These expressions led to the conditions $M\neq1$, $\left.Glu_{N\to E}\right|_{b}\neq0$ and $\left.V_{mg}>\left(1-M\right)Glu_{N\to E}\right|_{b}$ on the maximum rate of glutamate uptake determined the value $W=18.46$. We obtained $\left.Glu_{N\to E}\right|_{b}=0.147\mu M.s^{-1}$ and thus $\left.Glu_{E}\right|_{b}=2\mu M$. Note that this value corresponds to the range $1.1-10.4\mu M$ found in the literature .

Equally to the glutamate release, the steady state of the GABA release is:

$\left.GABA_{N\to E}\right|_{b}=\frac{Z}{z_{2}}c(z_{1},z_{2})\left.FR_{IN}\right|_{b}$ **(7)**

The stationary states of the extracellular GABA concentration and the GABA release are related by the 2^nd^ order equation:

$\begin{matrix} \left.\left(V_{m1}+V_{m3}-\left.GABA_{N\to E}\right|_{b}\right)GABA_{E}\right|_{b}^{2} \\ +\left.\left(K_{m1}V_{m3}+K_{m3}V_{m1}-K_{m1}\left.GABA_{N\to E}\right|_{b}-K_{m3}\left.GABA_{N\to E}\right|_{b}\right)GABA_{E}\right|_{b} \\ -K_{m1}K_{m3}\left.GABA_{N\to E}\right|_{b}=0 \end{matrix}.$ **(8)**

Choosing the baseline value $\left.GABA_{E}\right|_{b}=1000\mu M$ (S1 Table) in accordance to the range $530-1627\mu M$ found in the literature , we obtained $\left.GABA_{N\to E}\right|_{b}=6.863\mu M.s^{-1}$ for the baseline of the GABA release and thus $Z=613$ through Eq. (7) of this file. With these expressions, one has access to the normalization variable $norm_{u2}$ in the astrocytes flow contribution of Eq. (20) via the expression $norm_{u2}=\left.Glu_{E\to A}\right|_{b}+\left.GABA_{E\to A}\right|_{b}$.

Besides, the rates $V_{gme}$ and $V_{gba}$ (constant values) of glutamate and GABA degradation, respectively, can be deduced from the stationary states calculated in the previous section and are:

$V_{gme}=\left(1-M\right)\left.Glu_{N\to E}\right|_{b}$ **(9)**

and

$V_{gba}=\frac{V_{m3}}{\left.K_{m3}+GABA_{E}\right|_{b}}\left.GABA_{E}\right|_{b}.$ **(10)**

1. Grimbert F, Faugeras O. Bifurcation analysis of Jansen's neural mass model. Neural computation. 2006;18(12):3052-68.

2. Hascup ER, Hascup KN, Stephens M, Pomerleau F, Huettl P, Gratton A, et al. Rapid microelectrode measurements and the origin and regulation of extracellular glutamate in rat prefrontal cortex. Journal of neurochemistry. 2010;115(6):1608-20.

3. Hascup KN, Hascup ER, Pomerleau F, Huettl P, Gerhardt GA. Second-by-second measures of L-glutamate in the prefrontal cortex and striatum of freely moving mice. The Journal of pharmacology and experimental therapeutics. 2008;324(2):725-31.

4. McLamore ES, Mohanty S, Shi J, Claussen J, Jedlicka SS, Rickus JL, et al. A self-referencing glutamate biosensor for measuring real time neuronal glutamate flux. Journal of neuroscience methods. 2010;189(1):14-22.

5. Lee M, McGeer EG, McGeer PL. Mechanisms of GABA release from human astrocytes. Glia. 2011;59(11):1600-11.

6. Patel AB, de Graaf RA, Mason GF, Rothman DL, Shulman RG, Behar KL. The contribution of GABA to glutamate/glutamine cycling and energy metabolism in the rat cortex in vivo. Proc Natl Acad Sci U S A. 2005;102(15):5588-93.

7. Patel AB, Rothman DL, Cline GW, Behar KL. Glutamine is the major precursor for GABA synthesis in rat neocortex in vivo following acute GABA-transaminase inhibition. Brain research. 2001;919(2):207-20.
