# Supplementary material for: A New Computational Model for Neuro-Glio-Vascular Coupling: Astrocyte Activation Can Explain Cerebral Blood Flow Nonlinear Response to Interictal Events
Source: PLoS One. 2016 Feb 5;11(2):e0147292. doi: 10.1371/journal.pone.0147292 (PMC4743967; doi:10.1371/journal.pone.0147292)
Supplement: S1 Table — State variables are specified, together with their initial value (stationary state). Parameter description and values are given in S1 Table. (°) Chosen from [54]. (°°) Chosen to be the average in the range 2070–2630 μM mentioned in [77]. (*) Values obtained by stationary state (baseline) calculation (S3 File). Input is p(t) of Eqs 1 and 2. The integration of this ODE system by numerical methods such as Runge-Kutta 4 leads to the simulation of the output of the model, the total cerebral blood inflow given by fin (t) = 0.8fA(t)+0.2fN(t). (DOC) [file pone.0147292.s008.doc]

| **variable name** | **initial value** | **1^st^ order ordinary differential equation or sigmoid** |
| --- | --- | --- |
| average excitatory post-synaptic potential of main cells $EPSP_{PC}(t)$ | $3.097mV$(*) | $\frac{dEPSP_{PC}}{dt}=s_{1}(t)$ |
| $s_{1}(t)$ | $0mV.s^{-1}$ | $\begin{matrix} \frac{ds_{1}}{dt}=Aap(t)+AaC_{PC\to PC}FR_{back}(t) \\ -2as_{1}(t)-a^{2}EPSP_{PC}(t) \end{matrix}$ |
| average inhibitory post-synaptic potential $IPSP_{PC}(t)$ | $3.348mV$(*) | $\frac{dIPSP_{PC}}{dt}=s_{2}(t)$ |
| $s_{2}(t)$ | $0mV.s^{-1}$ | $\begin{matrix} \frac{ds_{2}(t)}{dt}=BbC_{IN\to PC}FR_{IN}(t) \\ -2bs_{2}(t)-b^{2}IPSP_{PC}(t) \end{matrix}$ |
| average excitatory post-synaptic potential of interneurons $EPSP_{IN}(t)$ | $0.005mV$(*) | $\frac{dEPSP_{IN}}{dt}=s_{3}(t)$ |
| $s_{3}(t)$ | $0mV.s^{-1}$ | $\frac{ds_{3}}{dt}=AaFR_{PC}(t)-2as_{3}(t)-a^{2}EPSP_{IN}(t)$ |
| firing rate of the backward loop on main cells $FR_{back}(t)$ | $0.237s^{-1}$(*) | $FR_{back}(t)=\frac{2e_{0}}{1+\exp\left[r_{N}s_{N}-r_{N}C_{PC\to IN}EPSP_{IN}(t)\right]}$ |
| firing rate of main cells $FR_{PC}(t)$ | $0.147s^{-1}$(*) | $FR_{PC}(t)=\frac{2e_{0}}{1+\exp\left[r_{N}s_{N}-r_{N}\left(EPSP_{PC}(t)-IPSP_{PC}(t)\right)\right]}$ |
| firing rate of interneurons $FR_{IN}(t)$ | $0.207s^{-1}$(*) | $FR_{IN}(t)=\frac{2e_{0}}{1+\exp\left[r_{N}s_{N}-r_{N}C_{IN\to IN}EPSP_{IN}(t)\right]}$ |
| glutamate release in the extracellular space $Glu_{N\to E}(t)$ | $0.147\mu M.s^{-1}$(*) | $\frac{dGlu_{N\to E}}{dt}=s_{4}(t)$ |
| $s_{4}(t)$ | $0\mu M.s^{-2}$ | $\begin{matrix} \frac{ds_{4}}{dt}=Ww_{1}\exp\left(w_{2}\frac{\ln(w_{1}/w_{2})}{w_{1}-w_{2}}\right)FR_{PC}(t) \\ -(w_{1}+w_{2})s_{4}(t)-w_{1}w_{2}Glu_{N\to E}(t) \end{matrix}$ |
| extracellular glutamate concentration $Glu_{E}(t)$ | $2\mu M$(*) | $\frac{dGlu_{E}}{dt}=Glu_{N\to E}(t)-\frac{1}{1-M}\frac{V_{mg}}{1+\exp\left[r_{g}s_{g}-r_{g}Glu_{E}(t)\right]}$ |
| astrocytic glutamate concentration $Glu_{A}(t)$ | $1330\mu M$(°) | $\frac{dGlu_{A}}{dt}=\frac{V_{mg}}{1+\exp\left[r_{g}s_{g}-r_{g}Glu_{E}(t)\right]}-V_{gme}$ |
| GABA release in the extracellular space $GABA_{N\to E}(t)$ | $6.863\mu M.s^{-1}$(*) | $\frac{dGABA_{N\to E}}{dt}=s_{5}(t)$ |
| $s_{5}(t)$ | $0\mu M.s^{-2}$ | $\begin{matrix} \frac{ds_{5}}{dt}=Zz_{1}\exp\left(z_{2}\frac{\ln(z_{1}/z_{2})}{z_{1}-z_{2}}\right)FR_{IN}(t) \\ -(z_{1}+z_{2})s_{5}(t)-z_{1}z_{2}GABA_{N\to E}(t) \end{matrix}$ |
| extracellular GABA concentration $GABA_{E}(t)$ | $1000\mu M$(*) | $\begin{matrix} \frac{dGABA_{E}}{dt}=GABA_{N\to E}(t)-\frac{V_{m3}}{K_{m3}+GABA_{E}(t)}GABA_{E}(t) \\ -\frac{V_{m1}}{K_{m1}+GABA_{E}(t)}GABA_{E}(t) \end{matrix}$ |
| astrocytic GABA concentration $GABA_{A}(t)$ | $2320\mu M$(°°) | $\frac{dGABA_{A}}{dt}=\frac{V_{m3}}{K_{m3}+GABA_{E}(t)}GABA_{E}(t)-V_{gba}$ |
| neuronal contribution to the cerebral blood flow $f_{N}(t)$ | $1$ | $\frac{df_{N}}{dt}=s_{6}(t)$ |
| $s_{6}(t)$ | $0s^{-1}$ | $\frac{ds_{6}}{dt}=\frac{\varepsilon_{n}}{norm_{u1}}\left[EPSP_{PC}(t)-norm_{u1}\right]-\frac{1}{\tau_{sn}}s_{6}(t)-\frac{f_{N}(t)-1}{\tau{}_{fn}}$ |
| astrocytes contribution to the cerebral blood flow $f_{A}(t)$ | $1$ | $\frac{df_{A}}{dt}=s_{7}(t)$ |
| $s_{7}(t)$ | $0s^{-1}$ | $\begin{matrix} \frac{ds_{7}}{dt}=\frac{\varepsilon_{a}}{norm_{u2}}\left[\frac{V_{mg}}{1+\exp\left[r_{g}s_{g}-r_{g}Glu_{E}(t)\right]}+\frac{V_{m3}}{K_{m3}+GABA_{E}(t)}GABA_{E}(t)\right. \\ \left.-norm_{u2}\right]-\frac{1}{\tau_{sa}}s_{7}(t)-\frac{f_{A}(t)-1}{\tau{}_{fa}} \end{matrix}$ |
